# Between automatic and control processes: How relationships between problem elements interact to facilitate or impede insight

**DOI:** 10.3758/s13421-022-01277-3

**Published:** 2022-03-08

**Authors:** Maxi Becker, Simon Davis, Roberto Cabeza

**Affiliations:** 1 Humboldt University of Berlin, Department of Psychology, Unter den Linden 6, 10177 Berlin, Germany; 2 Duke University, Center for Cognitive Neuroscience, Durham, NC 27708 USA

**Keywords:** Insight, Problem solving, Remote associates task, Spreading activation, Cognitive control

## Abstract

Solving a problem requires relating the pieces of information available to each other and to the solution. We investigated how the strength of these relationships determines the likelihood of solving insight tasks based on remote associates. In these tasks, the solver is provided with several cues (e.g., *drop*, *coat*, *summer*) and has to find the solution that matches those cues (e.g., *rain*). We measured the semantic similarity between the cues and the solution (*cue–solution similarity*) as well as between cues (*cue–cue similarity*). We assume those relationships modulate two basic processes underlying insight problem-solving. First, there is an *automatic activation process* whereby conceptual activation spreads across a semantic network from each cue node to their associated nodes, potentially reaching the node of the solution. Thus, in general, the higher cue–solution similarity, the more likely the solution will be found (Prediction 1). Second, there is a *controlled search process* focused on an area in semantic space whose radius depends on competing cue–cue similarity. High cue–cue similarity will bias a search for the solution close to the provided cues because the associated nodes shared by both cues are highly coactivated. Therefore, high cue–cue similarity will have a beneficial effect when the cue–solution similarity is high but a detrimental effect when cue–solution similarity is low (Prediction 2). Our two predictions were confirmed using both verbal and pictorial remote association tasks, supporting the view that insight is dependent on an interaction of meaningful relationships between cues and solutions, and clarify the mechanisms of insight problem solving in remote associates.

**Supplementary Information:**

The online version contains supplementary material available at 10.3758/s13421-022-01277-3.

In today’s rapidly changing society, it is becoming increasingly important to solve complex problems that do not have an obvious solution, such as those involving insight. Although there are various definitions of insight, here, we define it as the sudden comprehension or solution of a nonobvious problem. Such solutions involve an AHA! experience (Danek et al., 2020, Kounios & Beeman, 2014), which refers to the solver’s conviction that the solution emerged suddenly, and is obviously and satisfyingly correct (Danek & Wiley, 2017; Topolinski & Reber, 2010). There are multiple factors determining the difficulty of insight tasks (Kershaw & Ohlsson, 2004). One key factor is how the problem is mentally represented by the solver based on the relationship between the key problem elements (i.e., the cues and the solution). In the current study, we investigate how the strength of these relationships determines the likelihood of solving these insight tasks.

## Automatic and control processes in RATs

One popular kind of insight problems is the remote associate task (RAT). In these tasks, participants are provided with a few pieces of information, or *cues*, and they have to find a solution word that is based on a conceptual link to all of the cues (Mednick, 1962, 1968). The most popular of the RATs is the Compound Remote Associate Task (C-RAT, Bowden & Jung-Beeman, 2003). Here, the cues are three words (e.g., *drop*, *coat*, *summer*) and the solution (e.g., *rain*) is a word that forms compounds with all three cues. According to dual-process models, two processes have been proposed contributing to RAT performance and creativity tasks in general: automatic activation processes and controlled processes (Beaty et al., 2015; Wiley & Jarosz, 2012; for reviews, see Barr, 2018; Benedek & Jauk, 2018). Here, we will argue that both processes determining insight performance in RATs are mediated by the relationships between cues and solution.

The *automatic* process is the spreading of activation across a semantic network of conceptually related information, in which concepts can be described as individual nodes that are linked in varying degrees of strength (or distance) by their conceptual similarity. This strength reflects how likely one node can activate another node. It is generally assumed that when a RAT problem is presented, the cues (e.g., *drop*, *coat*, *summer*) activate their corresponding nodes in a semantic network and activity spreads from these nodes to semantically associated nodes (e.g., water, jacket, winter; Becker, et al., 2020b; Holyoak, 1984). The spreading activation process is assumed to be unconscious and to continue automatically once it starts. If the converging spreading activity from the cues or their associates reaches the solution node and generates a level of activity that exceeds a certain threshold, the solution may “pop” into solver’s mind (Becker et al., 2020b, c; Bowers et al., 1990). This may explain why the solution often appears suddenly to the solver (Becker et al., 2020a). Several priming studies have provided evidence that automatic processes are important for RAT problem solving (e.g., Bowden, 1997; Bowden & Beeman, 1998; Howe et al., 2010).

In contrast, the *controlled* process involved in solving the RAT includes the inhibition of close associates and a memory search process (Badre et al., 2005). The inhibitory process is necessary because strongly activated close associates can interfere with finding the weakly activated remote associates that include the solution (Becker et al., 2020b, c; Gick & Halyoak, 1980). Consistent with this idea, Gupta et al. (2012) found that performance in RATs increases the better the solver can avoid retrieving high-frequency candidate solutions. Memory search refers to retrieving a specific content from memory. In contrast to automatic processing, a memory search is assumed to be a conscious, serial and effortful process requiring cognitive control (Badre et al., 2005; Kumar et al., 2021). Hence, this controlled process allows activating memory content over and beyond what is already activated due to spreading activation processes from the cues. Note, the amount of cognitive control required to retrieve content from memory varies strongly depending on how much it is already preactivated via automatic processes. Here, we will refer to memory search when talking about controlled processes. The memory search is less obvious in RATs than in a typical semantic memory retrieval task (Davelaar, 2015). As an example of the latter, if one is asked which animal living in the ocean is not a mammal, one could generate a few candidate animals living in the ocean (e.g., tuna, shark, dolphin) and then narrow the search by excluding incorrect answers (e.g., the tuna is a fish because it has gills and lays eggs) until the solution is found. In case of the RATs, it is difficult to organize a memory search because the topic of the answer is unknown. Whereas in the *ocean animal* example one knows the answer is a mammal’s name, in the C-RAT problem *drop/coat/summer*, it is not obvious that the solution is a liquid (rain). So how can the solver organize a memory search in such a situation?

Currently, there is little work exploring the search strategies in insight tasks such as RATs (Davelaar, 2015; Gupta et al., 2012; Smith et al., 2013). According to Smith et al. (2013), people use two search strategies to solve RATs: First, people produce candidate solutions primarily on the basis of just one of the three cues at a time. Second, people adopt a local search strategy—that is to say, they produce new candidate solutions based on their previous responses (Smith et al., 2013). Davelaar (2015) suggested that in addition to searching for candidate solutions that are closely related to each individual cue, participants also search for items that were automatically activated by the combination of all cues. According to the author, the reason for this is that the presentation of all three cues automatically activates an overlapping memory pattern or “semantic saliency map” that indirectly guides participants’ search behavior (Davelaar, 2015). This “semantic saliency map” describes the highest activations in the network caused by the cues. Note, although the precise interplay between automatic and controlled processes is still a matter of debate (for review, see Sowden et al., 2015, p. 16), Davelaar implies that the former (i.e., the cue related activation of a “semantic saliency map”) guides the latter (active search for candidate solutions). However, he assumes that the solution is *by definition* at the intersection of the cues’ semantic neighborhood and therefore likely the most salient point (i.e., most activated node) on this “semantic saliency map”. However, the author fails to address the differences in semantic similarity between cues, or between cues and a possible solution. For example, the cues (e.g., drop, coat, summer) may share close associates (e.g., winter) which are activated more strongly by the cues than the solution (rain) while the latter is only weakly related to the cues. As a result, the solution may not always be the most strongly activated node in the network (i.e., most salient point on this map). Thus, RAT performance may strongly depend on the semantic relationship between the cues and between the cues and the solution. The current study seeks to extend Davelaar’s model of insight problem solving in RATs to better explain how meaningful relationships between the problem elements influence insight performance.

## Influence of the relationship between problem elements on insight performance in RATs

We postulate that both *automatic* and *control* processes determining insight performance in RATs are mediated by the semantic relationship between the problem elements. Two types of relationships can be differentiated depending on the problem elements: (1) the strength of the relationship between the cues and the solution (cue–solution similarity), and (2) the strength of the relationship between the cues (cue–cue similarity).

**Fig. 1:**
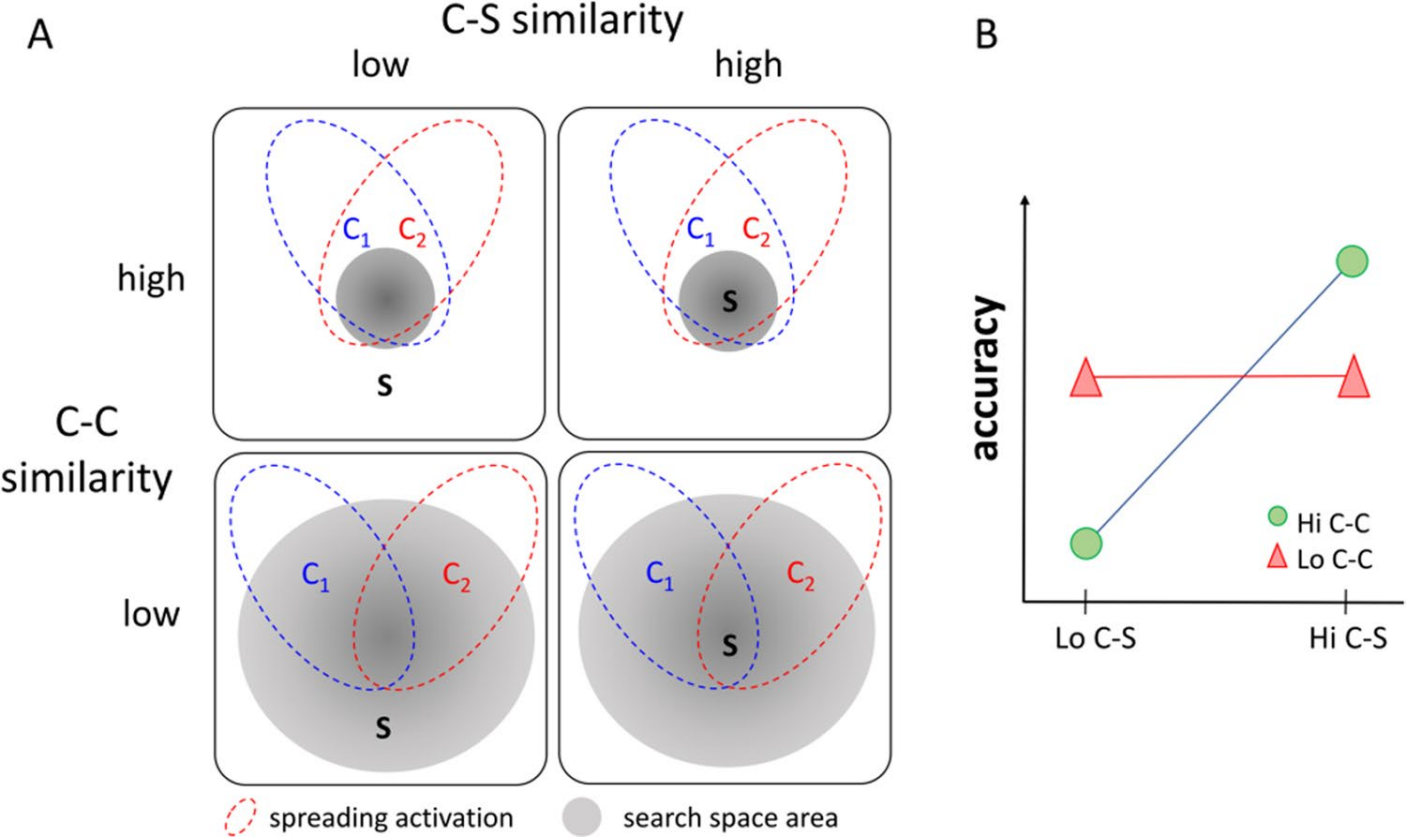
Schematic representation of influence of cue–solution and cue–cue similarity on RAT performance**.** *Note*. C_1_ = first cue; C_2_ = second cue; S = solution; C-C = cue–cue similarity; C-S = cue–solution similarity. This schema demonstrates the assumed interaction effect between cue–solution and cue–cue similarity on RAT performance (accuracy). We hypothesized that RAT performance should be mostly modulated by cue–solution similarity when the cue–cue similarity is high (Hi C-C; narrow search space) compared with when it is low (Lo C-C; wide search space; see Panel **b**)

*Cue–solution similarity* (e.g., drop↔rain, coat↔rain, and summer↔rain) determines how likely the solution will be automatically coactivated by the cues. We assume that when cue–solution similarity is high (right column in Fig. 1a), the solution is closer to the cues and more likely to lie within the area coactivated by the cues. In contrast, when cue–solution similarity is low (left column in Fig. 1a), the solution is farther away from the cues and less likely to be one of the nodes coactivated by the cues.

Cue–cue similarity (e.g., drop↔coat, drop↔summer, and coat↔summer) determines the “semantic saliency map” (i.e., how strongly the nodes are activated in the semantic network). When cue–cue similarity is high (upper row in Fig. 1a), the cues share more close associates, and hence, fewer nodes are activated in total but they are activated more strongly due to converging spreading of activity from multiple cues (Thagard & Stewart, 2011). In contrast, when cue–cue similarity is low, more nodes are activated in total but they are activated more weakly because they are associated with only a subset of the cues (lower row in Fig. 1a).

Importantly, we postulate that cue–cue similarity guides the memory search (controlled process). Specifically, the amount and strength of activated nodes in the semantic network will influence the size of the search space. The search space refers to all retrievable memory elements (concepts) that are related to the presented RAT problem. It defines the space in which the solver will conduct the memory search. Importantly, which memory elements are retrievable depends on the activation strength via automatic processes from the cues influencing the solver’s expectation of where to find the solution. When cue–cue similarity is high, the solver will expect the solution close to the cues due to the more strongly activated nodes around the cues (the most salient point on the “semantic saliency map” is centered around the cues). Subsequently, the solver will search for the solution more focally in the near semantic neighborhood of the cues. In contrast, when cue–cue similarity is low, the solver should not have an expectation of where to find the solution because many nodes are only weakly activated (there are no salient points on the saliency map). As a consequence, the solver will adopt a large search space and retrieve candidate solutions that may also be remotely related to the cues. Prior research shows that participants develop an expectation whether a RAT has a solution just based on brief exposure of the cues (Bolte & Goschke, 2005). This suggests that participants may use the relationship between the cues (cue–cue similarity) as a reference point of where to search for the solution.

Given those general assumptions, we make two *predictions* regarding the effects of cue–cue and cue–solution similarity on RAT performance.

*The first prediction is that independent of cue–cue similarity, RAT performance increases as a function of cue–solution similarity*. When cue–solution similarity is high, the solution is more likely to be automatically coactivated by multiple cues and the solution is reached faster by spreading activation from the cues (see second column of matrix in Fig. 1a).

*The second prediction is that cue–solution similarity additionally interacts with cue–cue similarity in predicting RATs performance.* Increasing cue–cue similarity has a positive effect on performance (i.e., increased accuracy, decreased solution time) with increasing cue–solution similarity because the solver will search focally in the close semantic neighborhood of the cues where the solution can also be found (see top right cell of Fig. 1a). In contrast, increasing cue–cue similarity has a negative effect on performance (i.e., decreased accuracy, increased solution time) with decreasing cue–solution similarity because the solver will search focally in the close semantic neighborhood of the cues when the solution is only weakly related to the cues (see top left cell of Fig. 1a). Performance is difficult to predict in the case when cue–cue similarity is low, because the search space is large and search is not guided by strongly activated nodes in the semantic network (see lower row of matrix in Fig. 1a). Eventually an intermediary performance can be expected compared with when cue–cue similarity high (see red vs. blue line in Fig. 1b). The reason for this is because the solver is neither guided to search in the area where the solution is (high cue–cue and high cue–solution similarity) nor misguided to search where the solution is not (high cue–cue and low cue–solution similarity; see upper row of matrix in Fig. 1a).

To test both predictions and demonstrate the generalizability of the results, we used two different versions of a RAT: an English C-RAT (Bowden & Jung-Beeman, 2003) and the novel language-independent RAT (LI-RAT; Becker & Cabeza, 2021). In the LI-RAT, the cues are two object pictures (e.g., corset, stopwatch), and the solution is an object (e.g., hourglass) that is perceptually associated to one cue (e.g., corset), but semantically associated to the other (e.g., stopwatch). Note, the LI-RAT has a conceptual but also a perceptual component. In both versions of the RAT, we measured and modeled the effects of conceptual cue–cue and cue–solution similarity on performance. Note, in the case of the LI-RAT, we additionally measured the effects of visual cue–cue and cue–solution similarity between items (based on qualitative ratings) on RAT performance. However, the latter was done for exploratory purposes and because solving the LI-RAT depends on a perceptual component. Insight performance was predicted by cue–cue and cue–solution similarity in both tasks. Accuracy and solution time served as objective measures for insight performance and the AHA! experience in addition to perceived suddenness of the solution served as subjective measures for insight performance. Davelaar (2015) only investigated objective measures of insight performance in RATs. However, Becker et al. (2020c) found a significant effect of cue–solution similarity on the AHA! experience in a modified version of the C-RAT (Becker et al., 2020c). For this reason, it is important to also explore subjective measures of insight when investigating RAT performance. However, Becker and colleagues did not correct for accuracy despite its strong correlation with the AHA! experience (Danek et al., 2014; Salvi et al., 2016; Webb et al., 2016). It is therefore unclear whether the similarities between the problem elements have an effect on the AHA! experience beyond their already assumed effect on accuracy.

## Methods

### Participants

The data for the present study was taken from an online sample of a previously published study (Becker & Cabeza, 2021). The purpose of this previous study was to introduce the LI-RAT as a language-independent creativity test by demonstrating significant interitem correlations between different language samples and providing its normed items as well as correlations to other creativity tests (e.g., the C-RAT). The current dataset comprises 183 English-speaking participants that had been recruited via the online platform Amazon Mechanical Turk. We excluded 21 subjects due to poor performance in either of the tasks (<10% accuracy). This resulted in a final sample of 162 English-speaking participants (age range: 22–69 years; 75 females: *M* = 44.1, 88 males: *M* = 38.5). The sample size was based on robust estimates for the normed LI-RAT items of the previously published study (*n* = 75 on average; Becker & Cabeza, 2021). All participants received a monetary compensation according to their time on task and the local ethics committee of the Humboldt University Berlin approved of the study.

### Materials and procedure

The online experiment was carried out via the research software Inquisit 4.0, and participants were tested individually (Draine, 1998). It consisted of three tasks: a language-independent remote associate task (henceforth LI-RAT), a compound remote associate task (henceforth C-RAT), and a short verbal fluency task (in this order). However, only the first two tasks are of relevance for the current research question.

#### C-RAT

In this task, participants are presented with three cue words (e.g., *drop*, *coat*, *summer*) and the goal of this task is to find a meaningful compound (e.g., *rain*) word that can be combined with each of the three cue words (*raindrop*, *summer rain*, *raincoat*). Twenty normed English C-RAT items of varying difficulty were taken from Bowden and Jung-Beeman (2003). The items were chosen such that difficulty was distributed uniformly from 10%–100% solution likelihood (based on the norms from Bowden & Jung-Beeman, 2003).

**Fig. 2:**
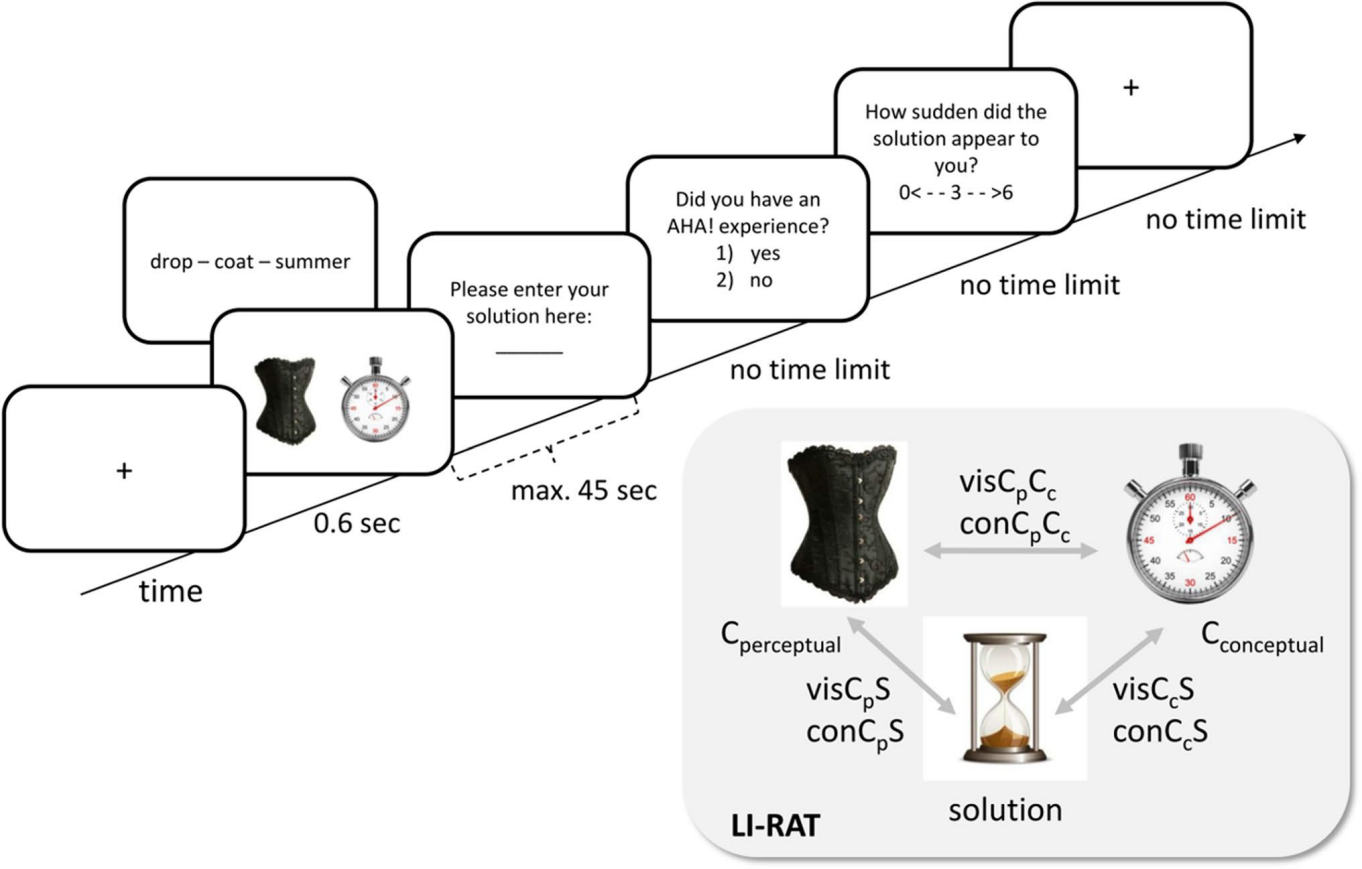
Time flow of the C-RAT and LI-RAT paradigm*. Note*. Upper panel: Participants were presented with C-RAT and LI-RAT problems for max. 45 seconds. Both tasks were identical in their procedure but were presented separately in two different experiments. When given a C-RAT problem, the participants were asked to find a compound word (rain) that can be put in front or in the back of all three cue words (drop–coat–summer). When given a LI-RAT problem, the participants were asked to find an object (hourglass) that connects the two seemingly unrelated presented objects (corset, stop watch). Upon solution, the participants were requested to enter their solution and rate their AHA! experience including how sudden the solution appeared to them. Lower panel: In the LI-RAT, the solution (S) is conceptually but not perceptually related to the stop watch (conceptual cue, C_c_) and perceptually but not conceptually related to the corset (perceptual cue, C_p_). Those relationships result in different visual and conceptual similarity pairs (see right panel) that were subsequently used to predict insight performance (see Table 1)

Before starting the experiment, participants completed two practice trials. The procedure is depicted in Fig. 2 (left panel) and looked as follows: The trial started with a fixation cross of 600 ms, followed by the presentation of three cue words on a white background for max. 45 s. The participants were instructed to press a key if they thought they had found the answer. If they did not press a key within 45 s, a new trial would start. If they pressed the solution key within those 45 s, they were subsequently prompted to type in their solution (no time limit). Finally, they were asked two follow-up questions regarding their subjective AHA! experience. For the present research, we concentrated on the perceived suddenness of and pleasure upon the solution that makes up an AHA! experience (see Danek et al., 2017), which was defined for the participants in the following way:

The AHA! experience is the feeling of pleasure when the solution came to you in a sudden manner. This can also be the case when you have already searched for the solution for quite some time. In contrast, the solution without insight appears to you in a more step-wise manner. For example, through active search you feel like you increasingly approached the solution.

The participants were first asked to rate whether they experienced an AHA! (yes/no answer). Because the definition of the AHA! experience is more strongly focused on the emotional response, we additionally asked to rate the perceived suddenness of the solution on a scale from 0 to 6 (both answers did not have a time limit). Finally, a new trial would start.

To summarize, the main variables of interest were the objective performance measures (accuracy and solution time). For exploratory reasons, we additionally assessed the subjective AHA! experience including suddenness. Accuracy reflects the amount of correctly solved C-RAT problems, and solution time describes the time from the problem presentation to the time of the solution button press.

#### LI-RAT

The LI-RAT was recently developed using pictures instead of words to extend the current set of remote associate tasks by a language-independent variant (see Becker & Cabeza, 2021). Similar to the C-RAT, item difficulty ranged between 8% and 95% (see Becker & Cabeza, 2021). Participants receive two cue pictures and need to come up with a third object (*target*) that is related to the two cues. One of the cues (*visual cue*) is always visually similar but conceptually dissimilar to the target, and the other cue (*conceptual cue*) is conceptually similar but visually dissimilar to the target. For example, when the participants are presented with a picture of a corset and a stopwatch, their task is to find the solution “hourglass” (see Fig. 2, right panel). The corset is visually similar to an hourglass due to its general hyperboloid shape and the stopwatch is conceptually similar to an hourglass because both objects measure time. The test is similar to the classic RAT in the sense that it requires thinking of the visual cue in an unusual way, focusing on its visual features (color, shape, etc.) rather than its meaning or function, in order to meaningfully relate both cues via a target object. The LI-RAT has a conceptual and a visual component but given our assumptions and predictions we were mostly interested in the conceptual component of the task. Importantly, in contrast to the C-RAT, the LI-RAT consists of only two instead of three cues. It is currently unknown whether the presentation of three cues interacts nonlinearly, biasing automatic and controlled processes. It is therefore also unknown whether simply averaging semantic similarities (as was done in the C-RAT) represents an adequate measure for cue–cue and cue–solution similarity. For this reason, it is important to present not only the results of the C-RAT but also the ones of the LI-RAT as a control measure and to demonstrate generalizability of the results.

The participants received 61 randomly chosen LI-RAT trials from a pool of 121 prevalidated items (see Becker & Cabeza, 2021). Participants were instructed to find a target object (hourglass) that was visually similar (but conceptually dissimilar) to one object (corset) on the screen and conceptually similar (but visually dissimilar) to the other object (stopwatch) on the screen. The amount of practice trials, the procedure and the timing were identical to the C-RAT with the exception that instead of three cue words two pictures were presented on white background (see Fig. 2, upper panel). The main variables of interest were the same as for the C-RAT (accuracy, solution time, AHA! experience including suddenness). Accuracy reflects the amount of correctly solved LI-RAT problems.

### Quantification of similarity between problem elements

Conceptual similarity between the problem elements of the C-RAT and the LI-RAT items was quantified via the shortest path length between interlinked word pairs (Fellbaum, 2005; Hamp & Feldweg, 1997; see below). To investigate the generalizability of this similarity measure, we additionally quantified conceptual similarity based on cosine similarity between two word pairs derived from a word embedding model (see Becker et al., 2020c). To quantify the visual component of the LI-RAT, we additionally quantified visual similarity between the problem elements via subjective ratings.

#### Conceptual similarity—Shortest path length in a lexical database

The underlying database for the C-RAT items was WordNet®, a large lexical English database whose words are grouped into sets of so-called cognitive synonyms (“synsets”) which represent a distinct concept (Fellbaum, 1998, 2005). Importantly, those 117 000 synsets form a network as they are interlinked via lexical and conceptual-semantic relationships.

The underlying database for the LI-RAT was GermaNet (Hamp & Feldweg, 1997). This German database is highly similar to WordNet®, as it also groups words that express the same semantic concept into synsets (144,113 in total). Both lexical data bases organize nouns into hierarchies of *is–a* relations (e.g., a cat *is an* animal. Here, conceptual similarity can be quantified based on information contained in this *is–a* hierarchy. For example, a *cat* might be considered more like a *dog* than a *house*, if *cat* and *dog* share *animal* as a common ancestor in an *is–a* hierarchy. Irrespective of the database, we quantified conceptual similarity between two words as the shortest path length between two synsets (s1, s2) to which the respective words belongs after Leacock and Chodorow (1998). This measure finds the shortest path between two concepts and scales that value by the maximum path length in the *is–a* hierarchy in which they occur. A high value signifies high similarity between two concepts. We chose this similarity measure as it is intuitive, widely used and, importantly, it can be equally calculated from the German and English database (the picture objects in the LI-RAT were available in German). Note, there are also other similarity measures based on path length (see Wu & Palmer, 1994).

#### Conceptual similarity (LI-RAT)—Cosine similarity from co-occurrences

To test the generalizability of the results, the conceptual similarity between the cues and the solution for the LI-RAT was additionally computed from statistical co-occurrences in text data via a preexisting word embedding model as previously published (Becker et al., 2020c^1^). This method is fundamentally different to the shortest path length in a lexical data base. Word embeddings represent words as dense numerical vectors derived from a neural-network that was trained a with huge text corpus using the word2vec algorithm (see Mikolov et al., 2013). The database for the current word embedding model comprised 72 million well-formed sentences from the Leipzig Corpora Collection sampled from news websites in German language and additional 1.4 million sentences from web texts (Biemann et al., 2007). The entire vocabulary of the word embedding model consisted of 455,050 words represented each in a 300-dimensional vector. For two words, the cosine similarity was defined as the angle between two (300-dimensional) vectors that represent those words in this vector space. Because the LI-RAT consists of pictures, before calculating the cosine similarity the pictures were first translated into words that best represent the objects in those pictures (see Becker & Cabeza, 2021). A sanity check revealed that the conceptual cue was conceptually more similar to the solution than the visual cue to the solution in the LI-RAT, *t*(121) = 4.13, *p* < .001, CI [0.03, 0.08].

#### Visual similarity—Human ratings

To quantify visual similarity between the cue pictures and the solution in the LI-RAT, we had 69 participants rate the visual similarity between those problem elements via the online platform Amazon Mechanical Turk. Due to premature termination of the experiment or incorrect execution of the task (one participant rated the conceptual instead of the visual similarity between the cues and the solution), we excluded data from eight participants which resulted in a final sample of 61 participants (mean age = 41.74 years, *SD* = 10.85, 27 females). The participants received 360 pairs of pictures (hence three picture pairs per item) in a randomized order. They were instructed to ignore the conceptual similarity between picture pairs and only rate their visual similarity on a scale from 0 (*no similarity*) to 100 (*very high similarity*). Finally, the similarity ratings were averaged across subjects for each picture. A sanity check revealed that the visual cues were rated visually more similar to the solution than the conceptual cues to the solution, *t*(121) = 20.69, *p* < .001, CI [36.06, 43.54].

### Analysis

To investigate the influence of similarity between the problem elements in both subjective and objective measures of insight performance in both tasks and both samples, we set up a series of (general) linear mixed models, (G)LMMs (see Tables S3–S8 in the Supplementary Material). All analyses were carried out in R (Version 4.0.4; R Core Team, 2014) using the lme4-package (Version 1.1-26; Bates et al., 2014) and the glmmTMB-package (Version 1.0.2.1; Brooks et al., 2017). The advantage of C-RAT and LI-RAT’s simple task structure is that for each individual problem the relationship between all problem elements (i.e., the cues and the solution) is known and can be modeled.

#### Modeling similarity between problem elements in the C-RAT

Because the solution is related to the cue words only via a conceptual (syntactic) relationship, we just modeled the conceptual cue–cue (∑C_1,2,3_) and cue–solution similarity (∑C_1,2,3_-S) as independent variables. Cue–cue similarity was quantified as the average shortest path length between all three cue–cue word pairs. Cue–solution similarity was defined as the average shortest path length of all three cue–solution word pairs. Mean conceptual cue–cue and cue–solution similarity values per item including difficulty can be viewed in Table S1 (Supplementary Material). We used the respective average values for the cue–cue and cue–solution similarity because we assumed that, in contrast to the LI-RAT, all cues are conceptually relevant to the solution. We hypothesized that the relationships between the problem elements interact. Therefore, we additionally modeled an interaction term between the cue–cue × cue–solution similarity (∑C_1,2,3_* ∑C_1,2,3_-S). When modeling the AHA! experience including suddenness in both tasks for exploratory reasons, we additionally added the covariate accuracy to the regression models. The AHA! experience is highly correlated with accuracy but we were only interested in the effect that the similarities between the problem elements had on the AHA! experience over and beyond accuracy (Salvi et al., 2016; Webb et al., 2016). Subjects were always modeled as random intercept (for a visual depiction of the regression model, see Table 1).

**Table 1:** (G)LMM: Influence of cue–cue and cue–solution similarity between problem elements onto performance and the AHA! experience in the C-RAT and LI-RAT

**C-RAT** performance ~ ∑C_1,2,3_* ∑C_1,2,3_-S + (1|subjects) + ε AHA! ~ ∑C_1,2,3_* ∑C_1,2,3_-S + accuracy + (1|subjects) + ε **LI-RAT** performance~ visC_p_-C_c_*visC_p_-S+ conC_c_-C_p_*conC_c_-S + conC_p_-S+visC_c_-S + (1|subjects) +ε AHA! ~ visC_p_-C_c_*visC_p_-S + conC_c_-C_p_*conC_c_-S + conC_p_-S + visC_c_-S + accuracy+ (1|subjects) +ε	

*Note.* We modeled performance (accuracy, solution time) and the AHA! experience including suddenness for both tasks using a (general) linear mixed model. Subjects served as random intercepts. Cue–cue and cue–solution similarity between the problem elements as fixed effects differed depending on the sample and the task. For the C-RAT, we modeled conceptual similarity between the cues (∑C_1,2,3_) and between the cues and the solution (∑C_1,2,3_-S) as well as their interaction (∑C_1,2,3_*∑C_1,2,3_-S). For the LI-RAT, we modeled conceptual and visual similarity between both cues and between the respective cue and the solution. visC_p_-C_c_ = visual similarity between both cues; conC_c_-C_p_ = conceptual similarity between both cues; visC_p_-S = visual similarity between the perceptually related cue and the solution; visC_c_-S = visual similarity between the conceptually related cue and the solution; conC_c_-S = conceptual similarity between the conceptually related cue and the solution; conC_p_-S = conceptual similarity between the perceptually related cue and the solution. In line with our hypotheses, an interaction between the solution relevant visual and conceptual cue–cue and cue–solution similarity was modeled (conC_c_-C_p_*conC_c_-S; visC_p_-C_c_*visC_p_-). The tilde signifies “predicted by”. ε = an error term

#### Modeling similarity between problem elements in the LI-RAT

Because the solution is related to the cue words via a conceptual *and* a visual relationship, we modeled both relationships separately. Hence, the conceptual similarity between (a) the conceptual and perceptual cue (conC_c_-C_p_), (b) both cues to the solution (conC_c_-S, conC_p_-S) and the visual similarity between (c) the conceptual and perceptual cue (visC_p_-C_c_), as well as (d) both cues to the solution (visC_c_-S, visC_p_-S) served as independent variables. Note, the visual similarity between the conceptually related cue to the solution (conC_p_-S) and the conceptual similarity between the perceptually related cue to the solution (visC_c_-S) were not solution relevant, according to the task instructions. Nonetheless, we modeled those relationships to account for the possible influence of cross-modal information. Because we hypothesized that the relationships between problem elements interact, we additionally modeled an interaction term between the cue–cue × cue–solution similarity for both modalities separately (visC_c_-C_p_ × visC_p_-S; conC_c_-C_p_ × conC_c_-S). All similarity values per item can be viewed in Table S2 (Supplementary Material). Note, item-wise difficulty values for the LI-RAT are reported in Becker and Cabeza (2021).

For both tasks, the dependent variables were the objective performance measures (accuracy and solution time) and, for exploratory reasons, the subjective AHA! experience including suddenness. Accuracy reflects the amount of correctly solved LI-RAT problems and solution time describes the time from the problem presentation till the solution button press. Accuracy and AHA! experience (both binary variables) were modeled assuming a binomial error distribution with the default logit link function (Bates et al., 2014). Solution time and suddenness were modeled assuming a Gaussian error distribution. To reach normality, solution time was log-transformed for both tasks. Visual inspection of the residual plots did not reveal obvious deviations from normality and homoscedasticity for log transformed solution time and suddenness. All independent variables were mean centered. To determine whether the interaction term between the respective cue–cue and cue–solution similarities was significant, we compared the models with and without the interaction term using likelihood ratio tests. If the interaction term was not significant, we report the respective model with the better fit to the data (without interaction). For the single predictors from those models, *p* values were obtained via the lmerTest-toolbox (Version 3.1-3) in R (Kuznetsova et al., 2017).

## Results

All parameter estimates of all models are reported in Tables S3–S8 in the Supplementary Material. For sake of brevity, only significant and nonsignificant results for the hypothesized main and interaction effects (conceptual cue–cue and cue–solution similarity) are reported for objective and subjective measures of insight.

### C-RAT

Participants correctly solved 79.0% (*SD* = 40.8%) of all presented C-RAT items in 7.11 seconds (*SD* = 8.90 seconds) on average. They reported to have experienced an AHA! upon solution for 61.1% (*SD* = 48.8%) of all solved items. Furthermore, on average they rated the perceived suddenness of the solution with 4.03 (*SD* = 2.10) on a scale from 0 (*not sudden but increasingly reached the solution*) to 6 (*very sudden solution*).

### Impact of conceptual similarities between problem elements on C-RAT performance

#### Accuracy

**Fig. 3:**
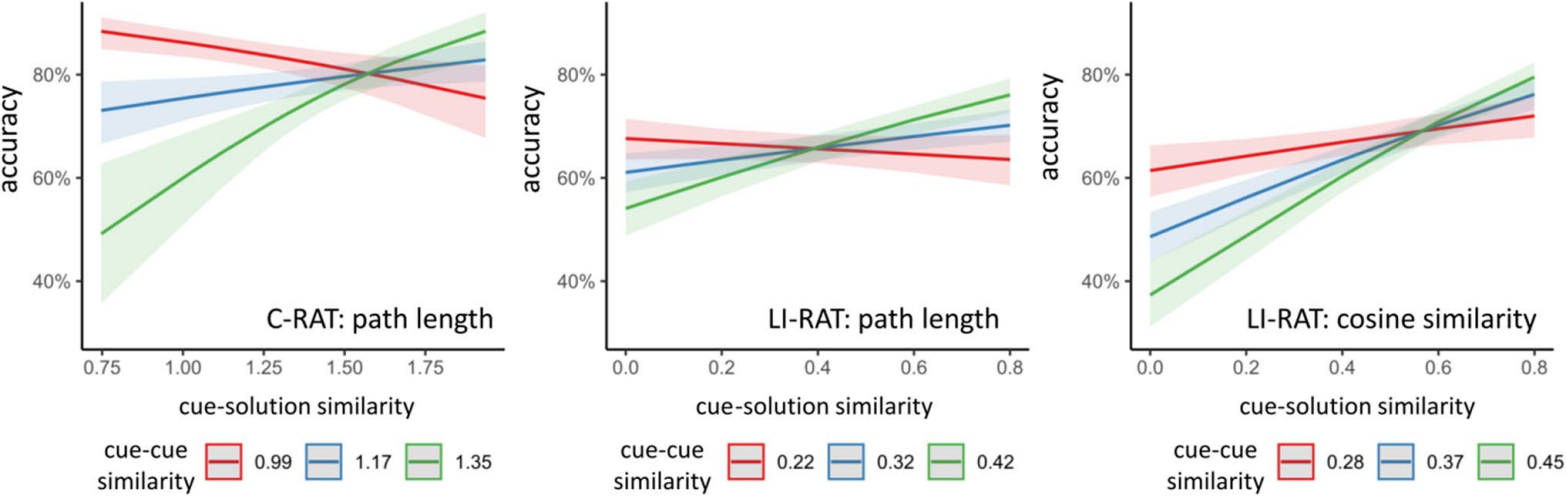
Interaction effect between conceptual cue–cue and cue–solution similarity on performance (accuracy) in C-RAT and LI-RAT. *Note*. Two different similarity measures were used for conceptual similarity in the LI-RAT. In the left and middle panel the conceptual similarity was quantified as shortest path length between two synsets. In the right panel, conceptual similarity was quantified as cosine similarity between two word vectors. As hypothesized insight performance as measured by accuracy increased with increasing cue–solution similarity. Additionally, accuracy was affected by an interaction between cue–solution similarity and cue–cue similarity. The colored shades around the lines represent the 95% confidence interval

Accuracy increases with cue–solution similarity (*z* = 2.19, *p* < .05) assuming medium cue–cue similarity. In contrast, accuracy decreased with increasing cue–cue similarity assuming medium cue–solution similarity (*z* = −5.65, *p* < .001). Importantly, the interaction term between cue–solution × cue–cue similarity was significant, χ^2^(1) = 24.04, *p* < .001). That is to say, the facilitating effect of cue–solution similarity on accuracy was heavily modulated by cue–cue similarity (see Fig. 3, left panel). As predicted, the positive relationship between cue–solution similarity and accuracy was strongest for high cue–cue similarity, while it became even negative for low cue–cue similarity.

#### Solution time

With increasing cue–solution similarity solution time drops significantly, *t*(2581.1) = −4.52, *p* < .001, assuming medium cue–cue similarity. In contrast, it takes participants significantly longer to reach the solution with increasing cue–cue similarity, *t*(2582.8) = 3.98, *p* < .001, assuming the medium cue–solution similarity. However, the interaction term between cue–solution similarity × cue–cue similarity was also a significant predictor of solution time, χ^2^(1) = 41.47, *p* < .001. Hence, the facilitating effect of increasing cue–solution similarity on solution time was again modulated by cue–cue similarity.

For an overview of the parameter estimates of both performance models, see Table S3 in the Supplementary Material.

### Impact of conceptual similarities between problem elements on C-RAT AHA! experience including suddenness

#### AHA! experience

When controlling for the impact of accuracy (*z* = 15.58, *p* < .001) on the amount of experienced AHA! upon solution, neither the cue–solution similarity (*p* > .60) nor the cue–cue similarity (*p* > .47) had an effect on the AHA! experience. There was also no evidence for a significant interaction effect between the cue–solution × cue–cue similarity (*p* > .08) in this model.

#### Suddenness

When controlling for accuracy, the strength of how sudden the solution word was perceived upon solution still increased with increasing cue–solution similarity, *t*(2546.3) = 4.36, *p* < .001) assuming medium cue–cue similarity. In contrast, the suddenness of the solution decreased with increasing cue–cue similarity, *t*(2547.4) = −2.47, *p* < .05 (assuming medium cue–solution similarity). Importantly, the cue-solution × cue–cue similarity interaction term was significant, χ^2^(1) = 36.03, *p* < .001. This means that when cue–cue similarity was high, the solution was perceived as increasingly more sudden with increasing cue–solution similarity compared with when cue–cue similarity was low. Additionally, the solution was perceived as significantly more sudden, when the solution was correctly compared with incorrectly solved, *t*(2669.7) = 26.15, *p* < .001. (For an overview of the parameter estimates of the AHA! experience and suddenness model, see Table S4 in the Supplementary Material.)

### LI-RAT

Participants correctly solved 65.7% (*SD* = 47.5%) of all presented LI-RAT items in 9.06 seconds (*SD* = 8.39 seconds) on average. They reported to have experienced an AHA! upon solution for 53.0% (*SD* = 49.9%) of all solved items. Furthermore, on average they rated the perceived suddenness of a solution with 3.70 (*SD* = 2.09) on a scale from 0 *(not sudden but increasingly reached the solution*) to 6 (*very sudden solution*).

### Impact of conceptual (shortest path length) and visual similarities between problem elements on LI-RAT performance

#### Accuracy

Assuming medium (average) conceptual cue–cue similarity, accuracy increased with increasing conceptual cue–solution similarity for the conceptual cue (conC_c_-S: *z* = 2.58, *p* < .05).

Assuming medium conceptual cue–solution similarity, there was no effect of cue–cue similarity on accuracy (*p* > .70). Importantly, the interaction term between conceptual cue-solution × cue–cue similarity was significant (conC_c_-S × conC_c_-C_p_), χ^2^(1) = 10.10, *p* < .01. That is to say, the positive relationship between cue–solution similarity and accuracy was strongest for high cue–cue similarity but this relationship vanished for low cue–cue similarity (see Fig. 3, middle panel).

#### Solution time

There was no evidence for an effect of conceptual cue–solution similarity on solution time assuming medium conceptual cue–cue similarity (conC_c_-S: *p* > .39). Solution time increased with increasing conceptual cue–cue similarity assuming medium conceptual cue–solution similarity (conC_c_-C_p_), *t*(8768.6) = 3.11, *p* < .01. There was no evidence for an interaction between the conceptual cue-solution cue–cue similarity (conC_c_-S × conC_c_-C_p,_ *p* > .98). Solution time further increased with decreasing (solution-irrelevant) conceptual cue–solution similarity of the irrelevant perceptual cue (conC_p_-S), *t*(8771.2) = −5.29, *p* < .001. Note, according to task instructions both cue-solution similarities (visC_c_-S, conC_p_-S) were not solution relevant. That is to say, participants were instructed that the perceptual cue is only visually but not conceptually related and the conceptual cue is only conceptually but not visually related to the solution. (For an overview of the parameter estimates of both performance models, see Table S5 in the Supplementary Material.)

### Impact of conceptual (cosine similarity) and visual similarities between problem elements on LI-RAT performance

#### Accuracy

Assuming medium (average) conceptual cue–cue similarity, accuracy increased with increasing conceptual cue–solution similarity for the conceptual cue (conC_c_-S: *z* = 7.37, *p* < .001) and decreasing (solution irrelevant) conceptual cue–solution similarity for the perceptual cue (conC_p_-S: *z* = −5.76, *p* < .001). Assuming medium conceptual cue–solution similarity, there was no effect of conceptual cue–cue similarity on accuracy (*p* > .17). Importantly, the interaction term between conceptual cue–solution × cue–cue similarity was significant (conC_c_-S × conC_c_-C_p_), χ^2^(1) = 10.08, *p* < .01. That is to say, the positive relationship between cue–solution similarity and accuracy was strongest for high cue–cue similarity but this relationship vanished for low cue–cue similarity (see Fig. 3, right panel).

#### Solution time

Solution time was significantly reduced with decreasing conceptual cue–solution similarity (conC_c_-S), *t*(8853.0) = −4.84, *p* < .001, and increasing conceptual cue–cue similarity (conC_c_-C_p_), *t*(8855.0) = 2.31, *p* < .05. There was no evidence for an interaction between the conceptual cue-solution × cue–cue similarity (conC_c_-S × conC_c_-C_p_: *p* > .46).

For an overview of the parameter estimates of both performance models, see Table S6 in the Supplementary Material.

### Impact of conceptual (path length) and visual similarities between problem elements on the LI-RAT AHA!-experience including suddenness

#### AHA! experience

When controlling for accuracy, there was no evidence for an effect of neither conceptual cue–solution similarity of the conceptual cue (concC_c_-S: *p* > .77), nor of conceptual cue–cue similarity (concC_c_-C_p_: *p*>.29) on the AHA! experience. Furthermore, there was no evidence for an interaction between the conceptual cue-solution × cue–cue similarity (conC_c_-S × conC_c_-C_p_: *p*>.21).

As expected, accuracy significantly increased the likelihood for experiencing an AHA! upon solving the LI-RAT (*z* = 30.02, *p* < .001). Additionally, increasing (solution irrelevant) conceptual cue–solution similarity of the perceptual cue increased the likelihood to experience an AHA! when solving a LI-RAT problem (concC_p_-S: *z* = 2.43, *p* < .05).

#### Suddenness

There was also no evidence for an effect of conceptual cue-solution of the conceptual cue (concC_c_-S: *p* > .57), nor cue–cue similarity (concC_c_-C_p_: *p* > .86) on the amount of perceived suddenness. Also no evidence for an interaction between the conceptual cue-solution × cue–cue similarity was observed (conC_c_-S × conC_c_-C_p_: *p* > .24). However, perceived suddenness increased with increasing (solution irrelevant) conceptual cue–solution similarity of the perceptual cue (concC_p_-S), *t*(8713.0) = 1.99, *p* < .05. As expected, when the LI-RAT problem was solved correctly, the solution was perceived as significantly more sudden compared with when it was not solved correctly, *t*(8832.0) = 43.74, *p* < .001.

For an overview of the parameter estimates of the AHA! experience and suddenness model, see Table S7 in the Supplementary Material.

### Impact of conceptual (cosine similarity) and visual similarities between problem elements on the LI-RAT AHA!-experience including suddenness

#### AHA! experience

When controlling for accuracy and assuming medium conceptual cue–cue similarity, conceptual cue–solution similarity of the conceptual cue increased the likelihood to experience an AHA! (conC_c_-S: *z* = 2.54, *p* < .05). Furthermore, the interaction between the conceptual cue–cue and cue–solution similarity was significant (conC_c_-S × conC_c_-C_p_), χ^2^(1) = 3.51, *p* < .001. Furthermore, accuracy significantly increased the likelihood for experiencing an AHA! upon solving the LI-RAT (*z* = 30.12, *p* < .001).

#### Suddenness

Assuming medium conceptual cue–cue similarity, increasing conceptual cue–solution similarity of the conceptual cue increased the experienced suddenness of the solution (conC_c_-S), *t*(8853.5) = 3.93, *p* < .001. In contrast, increasing conceptual cue–cue similarity decreased the experienced suddenness of the solution assuming medium conceptual cue–solution similarity of the conceptual cue (conC_c_-C_p_), *t*(8848.9) = −3.56, *p* < .001. The interaction between the conceptual cue–cue and cue–solution similarity of the conceptual cue was significant (conC_c_-S × conC_c_-C_p_), χ^2^(1) = 4.56, *p* < .05. Perceived suddenness increased with increasing (solution irrelevant) conceptual cue–solution similarity of the perceptual cue (concC_p_-S), *t*(8851.7) = 2.10, *p* < .05, and accuracy, *t*(8851.3) = 43.74, *p* < .001. (For an overview of the parameter estimates of the AHA! experience and suddenness model, see Table S8 in the Supplementary Material.)

## Discussion

Solving a problem requires relating pieces of meaningful information about the available clues to both each other and an eventual solution. This study extends Davelaar’s (2015) model of insight problem solving to better explain how the strength of the relationships between the cues (cue–cue similarity) *and* between the cues and the solution (cue–solution similarity) determines insight performance. We hypothesized that both automatic and control processes are mediated by the semantic relationships between the cues and the solution: cue–solution similarity determines how likely the solution will be automatically coactivated by the cues (automatic process), and result in a positive linear relationship between cue–solution similarity and insight performance (Prediction 1). Additionally, cue–cue similarity determines the size of the search space in which the solver will subsequently search for the solution (control process), resulting in an interaction between cue–cue and cue–solution similarity on insight performance (Prediction 2). Note, the search space depends on the activation strength via automatic processes from the cues influencing the solver’s expectation of where to find the solution: High cue–cue similarity is assumed to strongly activate few nodes due to converging spreading activity from multiple cues, which will lead the solver to expect and search for the solution in the near semantic neighborhood of the cues (narrow search space). Hence, performance should be particularly enhanced when cue–solution *and* cue–cue similarity is high because the solver will expect and search for the solution close to the cues where it is. In contrast, performance should be particularly low when cue–solution similarity is high but cue–cue similarity is low because the solver will expect and search for the solution to be close to the cues when it is not. Finally, low cue–cue similarity is assumed to activate many nodes, but only weakly because the cues do not share many related associates. As a consequence, the solver should not have a strong expectation of where to find the solution and search more broadly (large search space). Hence, performance is expected to be intermediate when cue–cue similarity is low because the solver’s search is neither directly guided towards nor away from the solution. Importantly, and in contrast to Davelaar’s (2015) model, we assume that the solver’s expectation of where to find the solution can be wrong because cues can be highly semantically related to each other (e.g., arm–leg–wing) but not necessarily to the solution (e.g., chair). We therefore predicted that cue–solution similarity additionally interacts with cue–cue similarity in determining insight performance (Prediction 2; see Fig. 1b).

We found evidence confirming both predictions using evidence from verbal and pictorial RATs demonstrating the generalizability of the results; these findings were stable specifically for objective measures of insight and irrespective of the type of similarity measure (path length or cosine distance). Those results are discussed below.

### Influence of conceptual cue–solution similarity and its interaction with conceptual cue–cue similarity on insight performance

Assuming medium cue–cue similarity, we found that increasing conceptual cue–solution similarity increases the likelihood to solve both RAT problems, irrespectively of how conceptual similarity was quantified (Prediction 1). The hypothesized positive relationship between cue–solution similarity and performance is congruent with previous priming studies assuming a facilitation effect of spreading activation on RAT performance (Bowden 1997; Bowden & Beeman, 1998; Howe et al., 2010). This relationship is further congruent with a study by Oltețeanu and Schultheiss (2019), who quantified cue–solution similarity using frequency (between word pairs from the Corpus of Contemporary American English) as a measure for associative strength instead of path length or cosine similarity. The authors also found a positive relationship between this measure and RAT performance, further stressing the relevance of automatic processes for this task (Oltețeanu & Schultheiss, 2019).

The positive effect of cue–solution similarity was additionally modulated by cue–cue similarity (Prediction 2). We found a significant interaction between conceptual cue–solution and cue–cue similarity predicting accuracy in both RAT problems and irrespective of how conceptual similarity was quantified. As predicted (see Fig. 1b), accuracy was highest when cue–solution and cue–cue similarity was high, and it was lowest when cue–solution similarity was low but cue–cue similarity was high. These results support our assumptions about how automatic and controlled search processes are mediated by the relationship between the cues and the solution in determining insight performance. A similar phenomenon relating low performance due to low cue–solution and high cue–cue similarity has been described by the Gestalt psychologists as the “fixation effect.” Here, the solver has difficulties solving a problem because she or he adheres to previous solutions that may not be useful in the current problem-solving context (Chrysikou & Weisberg, 2005; Duncker 1935/1945). Similarly, the solver may not search for more remotely related concepts of the cues because she or he is fixated on expecting the solution to be close to the cues. This fixation effect has been shown before in RATs. For example, Becker and colleagues (2020), Sio and colleagues (2017) as well as Smith and Blankenship (1991) report that RAT performance decreases when a distractor (e.g., a prime whose meaning is unrelated to the solution) is presented with the cues. Here, the solver likely adopts a too narrow search space, which tends to lead to a search in the semantic area of the distractor. Wiley (1998) reported a similar fixation effect with expert knowledge in RATs. Subjects with expert knowledge in a certain domain performed worse on a RAT if one of the three cues contained a word of their knowledge domain but required a different meaning, compared with control subjects without expert knowledge in that domain. Similar to the fixation effect due to the distractor, the author argued that the presentation of the respective cue automatically activates the knowledge structure narrowing the search space, preventing a broad search and ultimately decreasing the chances of finding the solution (Wiley, 1998). Wiley did not investigate cue–cue similarity in her study, but the size of the search space depended on prior knowledge (i.e., the amount of associations that the cue has to the rest of the concepts in the semantic network). As we did not specifically model this, future studies should further investigate what other factors determine the size of the search space (see Oltețeanu & Schultheiss, 2019). Importantly, what the present study clearly demonstrates is that the relationships between the different problem elements (specifically between cue–cue and cue–solution) interact with each other to determine insight performance and should therefore not be studied in isolation.

Additionally, similarity between the problem elements had reliable effects on subjective measures of insight performance (specifically suddenness), findings not central to our predictions but nonetheless relevant for problem solving. We discuss these and other findings below.

### Influence of cue–solution and cue–cue similarity on the AHA! experience

The exploratory results for the subjective AHA! experience were not as consistent as they were for performance specifically accuracy. This may be because accuracy was additionally modeled as a covariate of no interest, which, as expected, shared a significant amount of variance with the AHA! experience (Danek et al., 2014; Salvi et al., 2016; Webb et al., 2016). The perceived AHA! experience was not affected by any relationship between the problem elements including the solution after controlling for accuracy in the C-RAT and LI-RAT when conceptual similarity was modeled as the shortest path length. This indicates that the semantic relationship between the problem elements does not explain additional variance in the AHA! experience over and beyond what it already explains for accuracy.

The feeling of suddenness upon solution was significantly predicted by the relationship between the problem elements despite accuracy being a covariate in the model. This suggests that, in contrast to the general AHA! experience primarily representing the positive emotional response, perceived suddenness is not primarily caused by the correctness of the solution and therefore may reflect a different process compared with the positive emotional response. Danek and colleagues (2017) found that perceived suddenness upon solving magic tricks depended on the degree of how complex the solution was. That is to say, suddenness increased the less thinking steps participants had to take to solve this kind of insight task. Additionally, suddenness increased with increasing conceptual (and visual) cue–solution similarity in the C-RAT and LI-RAT of this study. Therefore, perceived suddenness may reflect the likelihood of the solution to be automatically coactivated by the problem elements and their close associates.

### Influence of cue–cue similarity on insight performance

Although this was not part of our predictions, we found a stable effect of cue–cue similarity on objective and subjective measures of insight for both domains. On average, increasing cue–cue similarity was associated with reduced accuracy, increased solution time, and reduced perceived suddenness upon solution (assuming medium conceptual cue–solution similarity). This negative impact on insight performance on average could be explained with a too narrow search space due to the solver’s expectation that the solution should be found close to the solution. Note, a reduced search space leads to increased performance, but only when the solution lies within this narrow space. However, on average, the solution is not in the very close semantic neighborhood of the cues, and therefore a broader search space would be required. This negative effect of cue–cue similarity on insight performance could also be explained with the aforementioned fixation effect reducing the size of the search space. Alternatively, the decrease in accuracy with increasing cue–cue conceptual similarity is reminiscent of the concept of “semantic confusability” in the object feature literature (see Clarke & Tyler, 2014). Semantic confusability describes the similarity of an object to its closest semantic neighbors and can be assessed quantitatively by the cosine between all overlapping in constituent feature vectors for pairs of object concepts in a given stimulus set. Further work in insight may therefore gain traction by applying such measures derived from the conceptual structure account of object concepts to help explain insight behaviors (see Taylor et al., 2007).

### Influence of visual cue–solution and cue–cue similarity on insight

Although we did not have a specific hypothesis for the visual domain, we found solid evidence for the predicted main effect (Prediction 1) for the visual domain: Increasing visual cue–solution similarity improved insight performance as measured by decreased solution time and increased accuracy as well as more AHA! experience and suddenness (see Tables S5–S8 in the Supplementary Material). This is plausible given that the solution is more likely to be coactivated by the cues and experienced suddenly by the solver the more associations the solution shares with the cues. However, in contrast to the conceptual domain, we did not find evidence that the interaction between the *visual* cue–solution and cue–cue similarity impacted performance in the LI-RAT (note, only when quantifying conceptual similarity as cosine distance, there was an interaction between the visual similarity of the problem elements in predicting the amount of perceived AHA! in the LI-RAT; see Table S8 in the Supplementary Material). This could imply that the insight problem is processed differently as a function of modality (visual vs. conceptual domain); conceptual solutions may depend on the interaction between the direct semantic link between a cue and solution in the context of competing alternatives; in contrast, visual similarity is much less prone to interference from competitors. Similarly, low to medium correlations between verbal and spatial/visual insight tasks (*r* = .20–.39) imply that there is still a significant amount of variance left unexplained in insight performance that may be in part due to the different modalities (see Chuderski & Jastrzębski, 2018). Alternatively, the stimuli in our task may have been too visually distinct to elicit strong interaction effects. Further research is required to investigate processing differences in insight between both modalities.

### Influence of “solution irrelevant” cue–solution similarity in LI-RAT

Interestingly and irrespective of modality, LI-RAT performance was not improved but impaired with increasing “solution-irrelevant” cue–solution similarity. “Solution-irrelevant” in this context refers to the conceptual similarity between the visually related cue and solution (conC_p_S) as well as visual similarity between the conceptually related cue and solution (visC_c_S). Those cue–solution similarities are “solution irrelevant” because the solver was instructed that the visual cue was not conceptually related and the conceptual cue was not visually related to the solution. Hence, the solver had the expectation that those “solution irrelevant” similarities would always be low, although this was not always the case.

Although one would assume that cue–solution similarity should generally improve performance, the reversed result in this case may be due to the solver’s expectation about the (instructed) relationship between problem elements including the solution. However, it demonstrates that cue–solution similarity does not automatically facilitate performance. That is to say, the network of the cues’ close associates does not always automatically activate the solution but rather when those associations are expected to be solution-relevant (as defined by task instructions for example). Although speculative but “solution-irrelevant” associations may get immediately inhibited by control processes and/or search is redirected to other regions of the search space. As mentioned before, the interplay between automatic and control processes is still largely unexplored (Barr et al., 2020). The results presented here indicate that automatic and control processes directly interact already during initial problem representation. Future research needs to further disentangle the precise relationship between both processes.

### Limitations

The insight tasks used for this study are different to *classic* insight tasks such as the nine-dot or eight-coin problem (Öllinger et al., 2013; Öllinger et al., 2014; Ormerod et al., 2002). Among other differences, those tasks are usually more difficult to solve, contain more problem elements and they are unique (there is only one exemplar) compared with the RAT tasks used in this study. For this reason, modeling the relationship between the cues and the solution is not possible with *classic* insight problems. Consequently, the results from this study cannot be automatically generalized to this type of insight tasks.

In general, cue–solution and cue–cue similarity combined explained only between 1.5% and 3.7% of the overall variance in RAT performance. Hence, although significant and systematic, those observed effects are rather small. Controlling for the amount of associations per cue (i.e., the amount of concepts associated with the cues) could have additionally reduced the amount of random noise in the model, increasing the amount of explained variance in RAT performance (see Oltețeanu & Schultheiss, 2019). However, small effect sizes were expected because problem difficulty in insight tasks is determined by multiple task-related and interindividual factors (see Kershaw & Ohlsson, 2004). Task-related factors are for example the conceptual and/or visual relationships between the cues and the solution as investigated here. Interindividual factors are for example prior knowledge, processing of the problem information and cognitive abilities of the solver. For example, word fluency ability has been shown to impact C-RAT and LI-RAT performance (Becker & Cabeza, 2021). This is consistent with the fact that the random intercept from the regression models investigated here representing inter-individual differences between subjects explained between 23% and 45% of the overall variance in insight performance (see Tables S3–S9 in the Supplementary Material).

## Conclusion

In this study, we investigated how the relationship between problem elements affect insight problem solving in RATs by mediating automatic and control processes. While insight performance is facilitated by cue–solution similarity (automatic processes) assuming medium cue–cue similarity, it generally depends on an interaction between both relationships at least for the conceptual domain. That is to say, the results suggest that participants use high cue–cue similarity as a heuristic that guides their memory search (control process), which can facilitate or impede insight performance depending on cue–solution similarity. Those results help clarifying the mechanisms of insight problem solving in remote associates.

## Supplementary Information

ESM 1 (DOCX 197 kb)

## Footnotes

1 Note, in this paper (Becker et al., 2020c) we published C-RAT norms with cosine distances (1- cosine similarity) and not similarity as this was easier to interpret given the former research question.
