# Supplementary material for: Between automatic and control processes: How relationships between problem elements interact to facilitate or impede insight
Source: Mem Cognit. 2022 Mar 8;50(8):1719–34. doi: 10.3758/s13421-022-01277-3 (PMC9767997; doi:10.3758/s13421-022-01277-3)
Supplement: Supplementary file 1 — (DOCX 197 kb) [file 13421_2022_1277_MOESM1_ESM.docx]

**Supplementary material**

| **Table 2.**  **List of LI-RAT items - cue-cue and cue-solution similarity and difficulty** | | | | | |
| --- | --- | --- | --- | --- | --- |
| C-RAT items | C-RAT solution | ∑C_1,2,3_ | ∑C_1,2,3_-S | accuarcy | RT |
| AGE-MILE-SAND | STONE | 1.30 | 1.32 | 0.49 | 13.28 |
| BOOT-SUMMER-GROUND | CAMP | 1.39 | 1.49 | 0.67 | 6.88 |
| CANE-DADDY-PLUM | SUGAR | 0.90 | 0.75 | 0.93 | 3.28 |
| COTTAGE-SWISS-CAKE | CHEESE | 1.13 | 1.94 | 0.97 | 2.3 |
| CREAM-SKATE-WATER | ICE | 1.25 | 1.66 | 0.98 | 2.01 |
| DATE-ALLEY-FOLD | BLIND | 1.04 | 1.61 | 0.67 | 11.1 |
| DRESS-DIAL-FLOWER | SUN | 1.19 | 1.26 | 0.85 | 6.43 |
| FOOD-FORWARD-BREAK | FAST | 1.16 | 1.32 | 0.92 | 5.16 |
| FRENCH-CAR-SHOE | HORN | 0.98 | 1.42 | 0.82 | 9.71 |
| MASTER-TOSS-FINGER | RING | 1.04 | 1.10 | 0.84 | 8.63 |
| MOUSE-BEAR-SAND | TRAP | 1.23 | 1.10 | 0.72 | 9.04 |
| PEACH-ARM-TAR | PIT | 0.99 | 1.12 | 0.72 | 8.26 |
| PIKE-COAT-SIGNAL | TURN | 1.44 | 1.40 | 0.63 | 10.67 |
| SAGE-PAINT-HAIR | BRUSH | 1.36 | 1.42 | 0.73 | 7.7 |
| SANDWICH-HOUSE-GOLF | CLUB | 0.90 | 1.03 | 0.82 | 8.15 |
| SLEEPING-BEAN-TRASH | BAG | 1.07 | 1.13 | 0.95 | 3.24 |
| WAGON-BREAK-RADIO | STATION | 1.21 | 1.65 | 0.46 | 15.87 |
| WATER-MINE-SHAKER | SALT | 1.53 | 1.52 | 0.91 | 5.4 |
| WORM-SHELF-END | BOOK | 1.18 | 1.10 | 0.92 | 3.9 |
| KNIFE-LIGHT-PAL | PEN | 1.18 | 1.34 | 0.78 | 7.42 |
| Note. ∑C_1,2,3_= conceptual mean cue-cue similarity (based on shortest path length); ∑C_1,2,3_-S = conceptual mean cue-cue similarity | | | | | |

| **Table 3.**  **List of LI-RAT items - cue-cue and cue-solution similarity and difficulty** | | | | | | | | | | | |
| --- | --- | --- | --- | --- | --- | --- | --- | --- | --- | --- | --- |
|  |  |  | similarity | | | | | | | | |
|  |  |  | pathlength | | | cosine | | | human rating | | |
| perceptual cue | conceptual cue | solution | conC_c_-C_p_ | conC_p_-S | conC_c_-S | conC_c_-C_p_ | conC_p_-S | conC_c_-S | visC_p_-C_c_ | visC_p_-S | visC_c_-S |
| TROLLEY | ICE CRYSTAL | SLEIGH | 0.31 | 0.55 | 0.31 | 0.31 | 0.31 | 0.3 | 8.11 | 53.97 | 8.54 |
| BAG | KEY | LOCK | 0.42 | 0.39 | 0.5 | 0.42 | 0.33 | 0.34 | 9.54 | 81.09 | 13.46 |
| BAT | GARLIC | VAMPIRE | 0.24 | 0.24 | 0.39 | 0.33 | 0.51 | 0.32 | 10.76 | 68.69 | 10.77 |
| BEER GLASS | PODIUM | TROPHY | 0.33 | 0.15 | 0.13 | 0.33 | 0.42 | 0.48 | 8.58 | 59.62 | 7.95 |
| CAULIFLOWER | CINE CAMERA | POPCORN | 0.26 | 0.46 | 0.31 | 0.28 | 0.64 | 0.48 | 8.41 | 75.63 | 9.51 |
| BOMB | FIRTREE | CHRISTMAS BAUBLE | 0.18 | 0.26 | 0.16 | 0.29 | 0.4 | 0.57 | 8.1 | 79.5 | 9.41 |
| BOOMERANG | STRAWBERRY | BANANA | 0.28 | 0.31 | 0.55 | 0.28 | 0.37 | 0.71 | 8.85 | 76.39 | 10.84 |
| BOWLING BALL | PALMTREE | COCONUT | 0.28 | 0.31 | 0.23 | 0.34 | 0.49 | 0.4 | 7.9 | 81.33 | 8.95 |
| BOWLING PINS | WINE GLASS | BOTTLE | 0.42 | 0.42 | 0.55 | 0.35 | 0.33 | 0.7 | 20.76 | 67.21 | 22.16 |
| BOWTIE | BUG | BUTTERFLY | 0.16 | 0.16 | 0.55 | 0.36 | 0.4 | 0.54 | 10.21 | 56.65 | 25.79 |
| BOXING | SUSHI | LOBSTER | 0.31 | 0.28 | 0.42 | 0.36 | 0.39 | 0.47 | 9.74 | 48.79 | 20.06 |
| BRANCH | DEER | ANTLERS | 0.26 | 0.39 | 0.23 | 0.35 | 0.5 | 0.42 | 20.49 | 66.44 | 63.31 |
| BRIDGE | WEATHER | RAINBOW | 0.23 | 0.26 | 0.61 | 0.3 | 0.39 | 0.36 | 9.48 | 51.95 | 15.62 |
| BROCCOLI | RADIOACTIVE | ATOMIC BOMB | 0.21 | 0.24 | 0.18 | 0.23 | 0.35 | 0.16 | 11.32 | 79.74 | 12.6 |
| IRONING BOARD | WETSUIT | SURFBOARD | 0.42 | 0.46 | 0.33 | 0.56 | 0.55 | 0.58 | 10.16 | 57.16 | 16.11 |
| BRUSH | LIPSTICK | MASCARA | 0.39 | 0.39 | 0.69 | 0.51 | 0.44 | 0.52 | 21.68 | 67.5 | 57.95 |
| FLATIRON | SALMON | SHIP | 0.19 | 0.39 | 0.19 | 0.39 | 0.39 | 0.31 | 36.5 | 66.73 | 45.95 |
| BULLET | SATELLITE | ROCKET | 0.36 | 0.36 | 0.39 | 0.32 | 0.54 | 0.44 | 13.48 | 70.98 | 19.95 |
| CAN | PLUNGER | TOILET | 0.39 | 0.46 | 0.42 | 0.4 | 0.34 | 0.37 | 13.07 | 56.08 | 9.89 |
| CAT | PYRAMID | SPHINX | 0.33 | 0.42 | 0.36 | 0.32 | 0.44 | 0.46 | 18.39 | 62.61 | 19.82 |
| CEILING FAN | CONCH | STARFISH | 0.21 | 0.19 | 0.5 | 0.35 | 0.51 | 0.43 | 10.57 | 62.89 | 33.95 |
| CELERY | SWING | SLIDE | 0.31 | 0.28 | 0.5 | 0.26 | 0.23 | 0.2 | 15.43 | 35.9 | 26.48 |
| CIGARETTE | CHALKBOARD | CHALK | 0.42 | 0.5 | 0.39 | 0.36 | 0.3 | 0.34 | 11.27 | 79.7 | 12.46 |
| CORSET | STOPWATCH | HOURGLASS | 0.36 | 0.36 | 0.69 | 0.32 | 0.32 | 0.54 | 9.23 | 40.85 | 12.23 |
| DALMATIAN | MILK | COW | 0.28 | 0.42 | 0.33 | 0.32 | 0.51 | 0.47 | 17.7 | 68.65 | 19 |
| TRIANGLE | NOTES | TRIANGLE | 0.19 | 0.18 | NaN | NaN | NaN | NaN | NaN | NaN | NaN |
| FERRISWHEEL | WHEAT | WATER MILL | 0.36 | 0.42 | 0.33 | 0.35 | 0.47 | 0.34 | 13.97 | 68.9 | 9.92 |
| FLOWER | VINYL | PHONOGRAPH | 0.36 | 0.33 | 0.36 | 0.4 | 0.38 | 0.54 | 9.85 | 66.34 | 28.51 |
| FOOTBALL | PATCH | SCAR | 0.39 | 0.26 | 0.26 | 0.33 | 0.26 | 0.4 | 17.84 | 60.52 | 35.03 |
| FILMCAN | CIRCUIT | BATTERY | 0.36 | 0.36 | 0.69 | 0.48 | 0.41 | 0.45 | 13.03 | 74.64 | 9.97 |
| FRIEDEGG | TAPE | CD | 0.39 | 0.39 | 0.69 | 0.4 | 0.39 | 0.73 | 17.64 | 59.87 | 21.43 |
| GASBOTTLE | FIRE | FIRE EXTINGUISHER | 0.24 | 0.39 | 0.21 | 0.38 | 0.58 | 0.37 | 11.4 | 78.13 | 13.72 |
| WATERING CAN | GIRAFFE | ELEPHANT | 0.16 | 0.19 | 0.46 | 0.36 | 0.41 | 0.69 | 53.59 | 52.79 | 36.45 |
| BELL | SCARF | HAT | 0.42 | 0.46 | 0.42 | 0.4 | 0.36 | 0.38 | 21.52 | 31.82 | 12.67 |
| GOLF BALL | ASTRONAUT | MOON | 0.18 | 0.31 | 0.23 | 0.31 | 0.4 | 0.29 | 18.18 | 73.42 | 15.87 |
| GRAPES | PARTY HAT | BALLOONS | 0.26 | 0.31 | 0.33 | 0.45 | 0.38 | 0.53 | 12.97 | 70.45 | 27.18 |
| GUN | COMB | HAIRDRYER | 0.5 | 0.61 | 0.55 | 0.3 | 0.42 | 0.39 | 18.4 | 83.27 | 23.56 |
| HANDCUFF | EYE | GLASSES | 0.31 | 0.42 | 0.28 | 0.32 | 0.38 | 0.37 | 19.85 | 66.76 | 18.77 |
| PHONE | SLIDERULE | CALCULATOR | 0.46 | 0.39 | 0.46 | 0.46 | 0.62 | 0.58 | 10.9 | 69.76 | 16.1 |
| HEDGEHOG | BARRETTE | BRUSH | 0.19 | 0.18 | 0.42 | 0.46 | 0.35 | 0.59 | 16.6 | 51.02 | 21.57 |
| ICE CONE | HEADPHONES | MICROPHONE | 0.46 | 0.42 | 0.61 | 0.44 | 0.42 | 0.6 | 9.56 | 75.31 | 13.7 |
| IGLOO | BASEBALL | BASEBALL CAP | 0.31 | 0.31 | 0.55 | 0.35 | 0.39 | 0.74 | 10.25 | 53.79 | 9.84 |
| INSECT | CAR | HELICOPTER | 0.19 | 0.19 | 0.46 | 0.39 | 0.39 | 0.54 | 8.21 | 68.79 | 15.92 |
| CABLE REEL | FILMTIMER | FILM REEL | NaN | 0.31 | NaN | 0.34 | 0.47 | 0.43 | 21.83 | 69.63 | 17.62 |
| NECKLACE | DOG | COLLAR | 0.31 | 0.55 | 0.31 | 0.27 | 0.48 | 0.34 | 12.65 | 52.35 | 9.51 |
| BUTTON | HOLE | GULLY | 0.26 | 0.39 | 0.31 | 0.47 | 0.38 | 0.46 | 46.81 | 66.62 | 58.7 |
| TIN | BABY | CRIB | 0.23 | 0.36 | 0.21 | 0.26 | 0.37 | 0.3 | 15 | 34.34 | 8.72 |
| CRAB | NAIL1 | PLIERS | 0.18 | 0.18 | 0.69 | 0.37 | 0.33 | 0.28 | 9.36 | 27.63 | 12.55 |
| LADDER | LOCO | RAILTRACK | 0.33 | 0.46 | 0.39 | 0.31 | 0.29 | 0.42 | 9.39 | 49.39 | 15.73 |
| DRAGONFLY | DOLL | FAIRY | 0.18 | 0.26 | 0.36 | 0.41 | 0.42 | 0.34 | 14.75 | 54.39 | 66.39 |
| LINDEN LEAF | CARD GAME | SPADE | 0.39 | 0.33 | 0.33 | 0.36 | 0.3 | 0.27 | 9.9 | 74.45 | 10.98 |
| RULER | POOL | DIVING BOARD | 0.42 | 0.46 | 0.5 | 0.32 | 0.35 | 0.31 | 16.63 | 48.36 | 42.49 |
| MAGNIFIER | POT | PAN | 0.42 | 0.42 | 0.69 | 0.37 | 0.41 | 0.64 | 17.97 | 72.23 | 24.67 |
| MAGNET | SHOES | HORSESHOE | 0.33 | 0.36 | 0.36 | 0.33 | 0.36 | 0.42 | 15.67 | 85.98 | 19.19 |
| SPIKY BALL | FISH | BLOWFISH | 0.23 | 0.19 | 0.69 | 0.45 | 0.55 | 0.55 | 10.52 | 52.31 | 59.59 |
| TAPE RULE | FROG | SNAIL | 0.19 | 0.23 | 0.39 | 0.38 | 0.41 | 0.64 | 15.18 | 65.94 | 31 |
| MICROWAVE | OSCAR | TV | 0.11 | 0.42 | 0.13 | 0.24 | 0.57 | 0.31 | 7.54 | 74.13 | 8.84 |
| MOSAIC | POSTER | PUZZLE | 0.5 | 0.46 | 0.42 | 0.47 | 0.63 | 0.39 | 40.9 | 57.31 | 38.25 |
| MARBLE | TRAMPOLINE | RUBBER BALL | 0.39 | 0.42 | 0.36 | 0.37 | 0.51 | 0.5 | 13.95 | 83.97 | 13.49 |
| SCREWNUT | BEE | HONEYCOMB | 0.14 | 0.36 | 0.21 | 0.5 | 0.53 | 0.45 | 8.44 | 21.25 | 15.03 |
| SLUG | COFFEE | CROISSANT | 0.21 | 0.23 | 0.36 | 0.43 | 0.43 | 0.51 | 21.79 | 77.44 | 22.26 |
| HOOK | SUCTION CUP | OCTOPUS | NaN | 0.19 | 0.19 | 0.6 | 0.5 | 0.54 | 36.05 | 48.71 | 14.86 |
| FOLDER | CLEF | ACCORDION | 0.24 | 0.36 | 0.24 | 0.39 | 0.36 | 0.33 | 17 | 69.8 | 19.97 |
| PAPER PILE | COIN | BANKNOTE | 0.33 | 0.33 | 0.61 | 0.36 | 0.35 | 0.54 | 9.65 | 41.15 | 10.66 |
| PEAR | CANDLE | LIGHT BULB | 0.36 | 0.33 | 0.69 | 0.49 | 0.6 | 0.54 | 16.15 | 68.4 | 19.6 |
| PEN | DRUMS | DRUMSTICKS | 0.33 | 0.31 | 0.61 | 0.16 | 0.49 | 0.43 | 8.95 | 57.08 | 10.23 |
| PENGUIN | CROSS | NUN | 0.15 | 0.24 | NaN | NaN | NaN | NaN | NaN | NaN | NaN |
| PEACOCK | VENTILATOR | FAN | 0.19 | 0.21 | 0.42 | 0.35 | 0.38 | 0.36 | 22.89 | 82.08 | 14.69 |
| MUSHROOM | CAGOULE | UMBRELLA | 0.31 | 0.36 | 0.39 | 0.38 | 0.32 | 0.59 | 14.34 | 61.9 | 8.13 |
| PISTACHIO | PEARLS | CLAM | 0.39 | 0.28 | 0.24 | 0.55 | 0.62 | 0.53 | 11.1 | 53.85 | 17.02 |
| POCKET WATCH | WORLD MAP | COMPASS | 0.5 | 0.69 | 0.5 | 0.3 | 0.32 | 0.41 | 41.54 | 83.77 | 42.36 |
| POLAROID | MAILBOX | STAMP | 0.46 | 0.26 | 0.26 | 0.31 | 0.45 | 0.47 | 8.84 | 61.26 | 9.42 |
| PORCUPINE | DUSTPAN | BROOM | 0.15 | 0.18 | 0.69 | 0.48 | 0.39 | 0.58 | 7.85 | 24.76 | 13.43 |
| PUMPKIN | TRAINERS | BASKETBALL | 0.33 | 0.31 | 0.36 | 0.37 | 0.35 | 0.41 | 6.86 | 82.41 | 7.15 |
| TIRE | HAND | RING | 0.36 | 0.42 | 0.39 | 0.25 | 0.32 | 0.27 | 7.39 | 66.25 | 7.63 |
| CLEANSER | BIRD | SWAN | 0.21 | 0.16 | 0.5 | 0.24 | 0.17 | 0.66 | 20 | 52.02 | 47.94 |
| LIFESAVER | MUFFIN | DOUGHNUT | 0.31 | 0.28 | 0.61 | 0.37 | 0.44 | 0.61 | 14.31 | 64.29 | 27.49 |
| PIPE SYSTEM | MOUSE | MAZE | 0.19 | 0.5 | 0.28 | 0.39 | 0.39 | 0.32 | 7.02 | 72.23 | 9.29 |
| ROOTS | BLOOD | VEINS | 0.42 | 0.33 | 0.31 | 0.32 | 0.34 | 0.38 | 19.52 | 68.39 | 36.08 |
| SALT SHAKER | RACKET | SHUTTLECOCK | 0.33 | 0.55 | 0.33 | 0.29 | 0.37 | 0.36 | 9.1 | 51.84 | 13 |
| BOX | MOTHERBOARD | LAPTOP | 0.46 | 0.46 | 0.55 | 0.44 | 0.51 | 0.61 | 32.46 | 71.77 | 29.65 |
| HOSE | POISON | SNAKE | 0.42 | 0.21 | 0.26 | 0.32 | 0.31 | 0.29 | 7.61 | 57.32 | 20.84 |
| SPONGE1 | COW | CHEESE | 0.26 | 0.31 | 0.33 | 0.34 | 0.41 | 0.44 | 9.14 | 76.9 | 12.59 |
| SPONGE2 | SHARPENER | ERASER | 0.39 | 0.39 | 0.69 | 0.51 | 0.51 | 0.71 | 48.5 | 54.94 | 31.76 |
| SOAP | MONITOR | COMPUTER MOUSE | 0.39 | 0.39 | 0.39 | 0.3 | 0.44 | 0.53 | 23.92 | 56.81 | 11.23 |
| SKI | NOODLES | CHOPSTICKS | 0.31 | 0.36 | 0.33 | 0.39 | 0.48 | 0.56 | 8.56 | 52.03 | 9.62 |
| SLEEPING BAG | EGYPT | MUMMY | 0.42 | 0.42 | 0.42 | 0.35 | 0.32 | 0.45 | 11.16 | 82.41 | 18.31 |
| SNAKE | PANTS | BELT | 0.21 | 0.21 | 0.55 | 0.31 | 0.27 | 0.39 | 8.56 | 70.9 | 8.02 |
| SPADE | CAKE | CAKE SERVER | 0.36 | 0.39 | 0.31 | 0.35 | 0.57 | 0.41 | 9.89 | 70.09 | 8.7 |
| COFFEE TABLE | FLY | SPIDER | 0.19 | 0.24 | 0.42 | 0.22 | 0.25 | 0.5 | 31.9 | 63.26 | 53.62 |
| NOODLE | BOTTLE OPENER | CORKSCREW | 0.31 | 0.31 | 0.69 | 0.48 | 0.45 | 0.69 | 15.84 | 44.57 | 37.87 |
| STAR FRUIT | SATURN | STAR | 0.42 | 0.42 | 0.69 | 0.3 | 0.32 | 0.5 | 18.69 | 51.27 | 10.19 |
| SOCKET | FACE MASK | FACE | 0.42 | 0.39 | 0.36 | 0.3 | 0.33 | 0.41 | 12.13 | 34.92 | 8.79 |
| PIN | CANDY | LOLLIPOP | 0.28 | 0.26 | 0.61 | 0.4 | 0.39 | 0.47 | 17.36 | 87.63 | 21.83 |
| STAMP | NAIL2 | HAMMER | 0.39 | 0.39 | 0.69 | 0.35 | 0.37 | 0.38 | 14.14 | 23.61 | 50.38 |
| STONE | STETHOSCOPE | HEART | 0.36 | 0.39 | 0.31 | 0.39 | 0.35 | 0.39 | 22.31 | 72.34 | 15.98 |
| STREET LIGHT | CROWN | SCEPTER | 0.46 | 0.42 | 0.5 | 0.32 | 0.28 | 0.4 | 25.05 | 68.48 | 21.1 |
| MATCH | BURGER | FRIES | 0.33 | 0.36 | 0.5 | 0.43 | 0.44 | 0.58 | 7.31 | 45.9 | 12.85 |
| T | SWEATPANTS | T-SHIRT | 0.23 | 0.21 | 0.42 | 0.31 | 0.25 | 0.6 | 12.93 | 37.19 | 23.56 |
| LAMP | BLANKET | TENT | 0.5 | 0.55 | 0.69 | 0.38 | 0.45 | 0.56 | 12.41 | 38.52 | 23.52 |
| TIEFIGHTER | WEIGHT BENCH | BARBELL | 0.36 | 0.36 | 0.69 | 0.47 | 0.4 | 0.58 | 26.3 | 69.24 | 23.15 |
| WHITEOUT | MOUSETRAP | MOUSE | 0.39 | 0.15 | 0.18 | 0.48 | 0.45 | 0.39 | 35.84 | 37.87 | 13.08 |
| PADDLE | CROSSWALK | STOP SIGN | 0.23 | 0.36 | 0.24 | 0.47 | 0.48 | 0.51 | 9.14 | 48.37 | 21.4 |
| EVIL EYE PERL | BINOCULARS | EYE | 0.28 | 0.28 | 0.28 | 0.43 | 0.33 | 0.38 | 15.68 | 63.03 | 12.94 |
| TOWER | FLASHLIGHT | LIGHT HOUSE | 0.5 | 0.81 | 0.46 | 0.35 | 0.51 | 0.31 | 41.48 | 72.33 | 52.74 |
| TV TOWER | PILLS | SYRINGE | 0.33 | 0.39 | 0.5 | 0.2 | 0.17 | 0.54 | 8.95 | 70.87 | 9.21 |
| TYRANNOSAUR | BABY SLING | KANGAROO | 0.21 | 0.46 | 0.21 | 0.6 | 0.49 | 0.45 | 10.73 | 65.03 | 31.23 |
| UFO | HOCKEY HELMET | PUCK | 0.36 | 0.46 | 0.42 | 0.44 | 0.33 | 0.5 | 17.79 | 42.03 | 22.75 |
| SPATULA | MOSQUITO | FLY SWATTER | 0.15 | 0.46 | 0.18 | 0.41 | 0.53 | 0.37 | 11.1 | 68.07 | 10.18 |
| VASE | COFFIN | URN | 0.55 | 0.55 | 0.69 | 0.45 | 0.43 | 0.67 | 12.54 | 64.71 | 17.51 |
| POWER STRIP | TURN SIGN | TRAFFIC LIGHTS | 0.39 | 0.39 | 0.36 | 0.41 | 0.31 | 0.48 | 8.95 | 67.49 | 12.3 |
| BIRDCAGE | GAVEL | PRISON | 0.46 | 0.42 | 0.33 | 0.26 | 0.38 | 0.17 | 10.18 | 54.82 | 8.78 |
| SCALE | PLAYGROUND | SEESAW | 0.42 | 0.42 | 0.55 | 0.39 | 0.45 | 0.58 | 22.46 | 62.87 | 20.46 |
| WALNUT | SKELETON | BRAIN | 0.42 | 0.39 | 0.42 | 0.38 | 0.37 | 0.47 | 21.39 | 67.9 | 10.79 |
| HELMET | CHAMELEON | TURTLE | 0.14 | 0.23 | 0.5 | 0.24 | 0.3 | 0.43 | 51.06 | 71.1 | 62.85 |
| WASHER | TRIPOD | CAMERA | 0.36 | 0.46 | 0.36 | 0.62 | 0.56 | 0.69 | 15.39 | 52.81 | 17.43 |
| BATTING | SUGAR SHAKER | COTTON CANDY | 0.39 | 0.36 | 0.42 | 0.34 | 0.51 | 0.46 | 25.89 | 63.41 | 51.41 |
| WHEEL | OVEN | PIZZA | 0.39 | 0.42 | 0.36 | 0.44 | 0.32 | 0.38 | 19.48 | 57.98 | 12.84 |
| ZEBRA | ROAD | CROSSWALK | 0.13 | 0.14 | 0.5 | 0.16 | 0.29 | 0.28 | 28.84 | 46.31 | 64.69 |
| SUGAR CANE | WHEELCHAIR | CANE | 0.26 | 0.28 | 0.42 | 0.28 | 0.34 | 0.54 | 8.43 | 29.2 | 11.08 |
| ONION DOME | CARROT | ONION | 0.33 | 0.36 | 0.61 | 0.38 | 0.44 | 0.72 | 14.23 | 60.56 | 28.89 |
| Note. visCp-S = visual similarity between perceptual cue and solution; visCp-Cc =visual cue-cue similarity; visCc-S = visual similarity between conceptual cue and solution; conCc-S =conceptual similarity between conceptual cue and solution; conCc-Cp = conceptual cue-cue similarity; conCp-S = conceptual similarity between perceptual cue and solution. | | | | | | | | | | | |

| **Table 4.** |  |  |  |  |  |  |  |  |  |
| --- | --- | --- | --- | --- | --- | --- | --- | --- | --- |
| **C-RAT: Impact of cue-cue & cue-solution similarity (path length) on accuracy and solution time** | | | | | | | | | |
|  | **accuracy** | | | | **log(solution time)** | | | | |
| Predictors | OR | 95% CI | z | p | std.β | 95% CI | t | df | p |
| (Intercept) | 3.66 | 3.14 – 4.27 | 16.56 | **<0.001** | 0.52 | 0.49 – 0.55 | 23.21 | 198.7 | **<0.001** |
| ∑C_1,2,3_-S | 1.14 | 1.01 – 1.28 | 2.19 | **0.029** | -0.05 | -0.07 – -0.02 | -4.52 | 2581.1 | **<0.001** |
| ∑C_1,2,3_ | 0.75 | 0.67 – 0.83 | -5.65 | **<0.001** | 0.03 | 0.01 – 0.04 | 3.98 | 2582.8 | **<0.001** |
| ∑C_1,2,3_-S:∑C_1,2,3_ | 1.40 | 1.22 – 1.61 | 4.80 | **<0.001** | -0.08 | -0.10 – -0.06 | -6.46 | 2580.2 | **<0.001** |
| Random Effects | | | | | | | | | |
| σ^2^ | 3.29 |  |  |  | 1.95 |  |  |  |  |
| ICC | 0.12 |  |  |  | 0.13 |  |  |  |  |
| Marg. R^2^ / Cond. R^2^ | 0.039 / 0.158 | |  |  | 0.017 / 0.147 | |  |  |  |
| *Note.* OR = Odds Ratio;Marg. R² = marginal R²; Cond. R² = conditional R²; ICC = intraclass coefficient; Std. β = standardized beta estimates; CI = confidence interval. ∑C_1,2,3_= conceptual mean cue-cue similarity (based on shortest path length); ∑C_1,2,3_-S = conceptual mean cue-cue similarity. | | | | | | | | | |
|  |  |  |  |  |  |  |  |  |  |
| **Table 5.** |  |  |  |  |  |  |  |  |  |
| **C-RAT: Impact of cue-cue & cue-solution similarity (path length) on AHA! experience including suddenness** | | | | | | | | | |
|  | **AHA!** | | | | **suddenness** | | | | |
| Predictors | OR | 95% CI | z | p | std.β | 95% CI | t | df | p |
| (Intercept) | 0.09 | 1.42 – 2.76 | -9.40 | **<0.001** | -0.07 | -0.14 – 0.01 | 11.09 | 803.8 | **<0.001** |
| accuracy | 32.57 | 2.70 – 3.59 | 15.58 | **<0.001** | 0.44 | 0.41 – 0.47 | 26.15 | 2669.7 | **<0.001** |
| ∑C_1,2,3_-S | 0.97 | 0.86 – 1.09 | -0.51 | 0.607 | 0.09 | 0.05 – 0.13 | 4.36 | 2546.3 | **<0.001** |
| ∑C_1,2,3_ | 0.96 | 0.85 – 1.08 | -0.71 | 0.478 | -0.04 | -0.08 – -0.01 | -2.47 | 2547.4 | **0.013** |
| ∑C_1,2,3_-S:∑C_1,2,3_ |  |  |  |  | 0.13 | 0.09 – 0.17 | 6.02 | 2546.3 | **<0.001** |
| Random Effects | | | | | | | | | |
| σ^2^ | 3.29 |  |  |  | 2.82 |  |  |  |  |
| ICC | 0.54 |  |  |  | 0.22 |  |  |  |  |
| Marg. R^2^ / Cond. R^2^ | 0.154 / 0.610 | |  |  | 0.203 / 0.379 | |  |  |  |
| *Note.* OR = Odds Ratio*;* Marg. R² = marginal R²; Cond. R² = conditional R²; ICC = intraclass coefficient; Std. β = standardized beta estimates; CI = confidence interval. ∑C_1,2,3_= conceptual mean cue-cue similarity (based on shortest path length); ∑C_1,2,3_-S = conceptual mean cue-cue similarity. | | | | | | | | | |

| **Table 6.** |  |  |  |  |  |  |  |  |  |
| --- | --- | --- | --- | --- | --- | --- | --- | --- | --- |
| **LI-RAT: Impact of cue-cue & cue-solution similarity (path length) on accuracy and solution time** | | | | | | | | | |
|  | **accuracy** | | | | **log(solution time)** | | | | |
| Predictors | OR | 95% CI | *z* | *p* | std. β | 95% CI | *t* | df | *p* |
| (Intercept) | 2.05 | 1.82 – 2.31 | 11.89 | **<0.001** | 0.65 | 0.62 – 0.67 | 49.88 | 1576 | **<0.001** |
| visC_p_-S | 1.04 | 0.99 – 1.10 | 1.74 | 0.082 | -0.02 | -0.03 – -0.01 | -6.44 | 8713 | **<0.001** |
| visC_p_-C_c_ | 0.80 | 0.76 – 0.85 | -7.70 | **<0.001** | 0.03 | 0.02 – 0.04 | 7.01 | 8713 | **<0.001** |
| visC_c_-S | 0.88 | 0.83 – 0.93 | -4.40 | **<0.001** | 0.01 | 0.00 – 0.02 | 2.79 | 8714 | **0.005** |
| conC_c_-S | 1.07 | 1.01 – 1.12 | 2.58 | **0.012** | -0.00 | -0.01 – 0.00 | -0.85 | 8714 | 0.397 |
| conC_c_-C_p_ | 0.99 | 0.93 – 1.05 | -0.35 | 0.703 | 0.01 | -0.00 – 0.02 | 3.11 | 8716 | **0.002** |
| conC_p_-S | 1.04 | 0.98 – 1.12 | 1.27 | 0.203 | -0.02 | -0.03 – -0.01 | -5.29 | 8716 | **<0.001** |
| conC_c_-S:conC_c_-C_p_ | 1.09 | 1.03 – 1.15 | 3.16 | **0.002** |  |  |  |  |  |
| Random Effects |  |  |  |  |  |  |  |  |  |
| σ^2^ | 3.29 |  |  |  | 0.85 |  |  |  |  |
| ICC | 0.13 |  |  |  | 0.18 |  |  |  |  |
| Marg. R^2^ / Cond. R^2^ | 0.027 / 0.153 | |  |  | 0.018 / 0.194 | |  |  |  |
| Note. OR = Odds Ratio; Marg. R² = marginal R²; Cond. R² = conditional R²; ICC = intraclass coefficient; Std. β = standardized beta estimates; CI = confidence intervall; log(solution time) = natural logarithm of solution time; visCp-S = visual similarity between perceptual cue and solution; visCp-Cc =visual cue-cue similarity; visCc-S = visual similarity between conceptual cue and solution; conCc-S =conceptual similarity between conceptual cue and solution; conCc-Cp = conceptual cue-cue similarity; conCp-S = conceptual similarity between perceptual cue and solution. | | | | | | | | | |

| **Table 7.** |  |  |  |  | |  |  | |  | |  | |  | |
| --- | --- | --- | --- | --- | --- | --- | --- | --- | --- | --- | --- | --- | --- | --- |
| **LI-RAT: Impact of cue-cue & cue-solution similarity (cosine similarity) on accuracy and solution time** | | | | | | | | | | | | | | |
|  | **accuracy** | | | | **log(solution time)** | | | | | | | | | |
| Predictors | OR | 95% CI | *z* | p | std. β | | | 95% CI | | t | | df | | p |
| (Intercept) | 2.04 | 1.81 – 2.30 | 11.74 | **<0.001** | 0.65 | | | 0.62 – 0.67 | | 50.00 | | 1576 | | **<0.001** |
| visC_p_-S | 1.11 | 1.06 – 1.16 | 4.24 | **<0.001** | -0.02 | | | -0.03 – -0.02 | | -7.37 | | 8855 | | **<0.001** |
| visC_p_-C_c_ | 0.79 | 0.74 – 0.83 | -8.87 | **<0.001** | 0.03 | | | 0.03 – 0.04 | | 7.40 | | 8856 | | **<0.001** |
| visC_c_-S | 0.89 | 0.85 – 0.94 | -4.24 | **<0.001** | 0.01 | | | 0.00 – 0.02 | | 2.79 | | 8857 | | **0.005** |
| conC_c_-S | 1.21 | 1.15 – 1.27 | 7.37 | **<0.001** | -0.03 | | | -0.03 – -0.02 | | -4.84 | | 8853 | | **<0.001** |
| conC_c_-C_p_ | 0.96 | 0.91 – 1.02 | -1.36 | 0.175 | 0.01 | | | 0.00 – 0.02 | | 2.31 | | 8855 | | **0.021** |
| conC_p_-S | 0.86 | 0.82 – 0.90 | -5.76 | **<0.001** | -0.00 | | | -0.01 – 0.01 | | -0.46 | | 8854 | | 0.644 |
| conC_c_-S:conC_c_-C_p_ | 1.08 | 1.03 – 1.13 | 3.15 | **0.002** |  | | |  | |  | |  | |  |
| Random Effects |  |  |  |  |  | | |  | |  | |  | |  |
| σ^2^ | 3.29 |  |  |  | 0.85 | | |  | |  | |  | |  |
| ICC | 0.13 |  |  |  | 0.18 | | |  | |  | |  | |  |
| Marg. R^2^ / Cond. R^2^ | 0.037 / 0.162 | |  |  | 0.018 / 0.195 | | | | |  | |  | |  |
| Note. OR = Odds Ratio; Marg. R² = marginal R²; Cond. R² = conditional R²; ICC = intraclass coefficient; Std. β = standardized beta estimates; CI = confidence intervall; log(solution time) = natural logarithm of solution time; visCp-S = visual similarity between perceptual cue and solution; visCp-Cc =visual cue-cue similarity; visCc-S = visual similarity between conceptual cue and solution; conCc-S =conceptual similarity between conceptual cue and solution; conCc-Cp = conceptual cue-cue similarity; conCp-S = conceptual similarity between perceptual cue and solution. | | | | | | | | | | | | | | |

| **Table 8.** |  |  |  |  |  |  |  |  |  |
| --- | --- | --- | --- | --- | --- | --- | --- | --- | --- |
| **LI-RAT: Impact of cue-cue & cue-solution similarity (path length) on AHA! experience including suddenness** | | | | | | | | | |
|  | **AHA!** | | | | **suddenness** | | | | |
| Predictors | OR | 95% CI | *z* | *p* | std.β | 95% CI | *t* | df | *p* |
| (Intercept) | 0.28 | 0.22 – 0.35 | -10.69 | **<0.001** | -0.01 | -0.08 – 0.06 | 30.48 | 2226 | **<0.001** |
| visC_p_-S | 1.13 | 1.07 – 1.19 | 4.53 | **<0.001** | 0.04 | 0.02 – 0.06 | 4.56 | 8711 | **<0.001** |
| visC_p_-C_c_ | 0.91 | 0.85 – 0.97 | -2.91 | **0.004** | -0.03 | -0.05 – -0.01 | -2.85 | 8711 | **0.004** |
| visC_c_-S | 1.05 | 0.98 – 1.12 | 1.32 | 0.187 | -0.01 | -0.03 – 0.02 | -0.57 | 8712 | 0.566 |
| conC_c_-S | 0.99 | 0.94 – 1.05 | -0.28 | 0.779 | -0.01 | -0.02 – 0.01 | -0.56 | 8711 | 0.573 |
| conC_c_-C_p_ | 1.04 | 0.97 – 1.11 | 1.05 | 0.292 | 0.00 | -0.02 – 0.02 | 0.17 | 8714 | 0.862 |
| conC_p_-S | 1.08 | 1.02 – 1.16 | 2.43 | **0.015** | 0.02 | 0.00 – 0.04 | 1.99 | 8713 | **0.046** |
| accuracy | 7.26 | 6.38 – 8.26 | 30.02 | **<0.001** | 0.42 | 0.40 – 0.44 | 43.74 | 8832 | **<0.001** |
| Random Effects |  |  |  |  |  |  |  |  |  |
| σ^2^ | 3.29 |  |  |  | 2.97 |  |  |  |  |
| ICC | 0.36 |  |  |  | 0.20 |  |  |  |  |
| Marg. R^2^ / Cond. R^2^ | 0.146/ 0.449 |  |  |  | 0.176 /0.340 |  |  |  |  |
| Note. OR = Odds Ratio; Marg. R² = marginal R²; Cond. R² = conditional R²; ICC = intraclass coefficient; Std. β = standardized beta estimates; CI = confidence intervall; log(solution time) = natural logarithm of solution time; visCp-S = visual similarity between perceptual cue and solution; visCp-Cc =visual cue-cue similarity; visCc-S = visual similarity between conceptual cue and solution; conCc-S =conceptual similarity between conceptual cue and solution; conCc-Cp = conceptual cue-cue similarity; conCp-S = conceptual similarity between perceptual cue and solution. | | | | | | | | | |
|  |  |  |  |  |  |  |  |  |  |
| **Table 9.** |  |  |  |  |  |  |  |  |  |
| **LI-RAT: Impact of cue-cue & cue-solution similarity (cosine similarity) on AHA! experience including suddenness** | | | | | | | | | |
|  | **AHA!** | | | | **suddenness** | | | | |
| Predictors | OR | 95% CI | *z* | *p* | std.β | 95% CI | t | df | *p* |
| (Intercept) | 0.27 | 0.21 – 0.34 | -10.88 | **<0.001** | -0.02 | -0.08 – 0.05 | 30.30 | 224.0 | **<0.001** |
| visC_p_-S | 1.17 | 1.11 – 1.24 | 5.82 | **<0.001** | 0.05 | 0.03 – 0.06 | 5.00 | 8973.1 | **<0.001** |
| visC_p_-C_c_ | 0.92 | 0.87 – 0.99 | -2.41 | **0.016** | -0.02 | -0.05 – -0.00 | -2.31 | 8852.0 | **0.021** |
| visC_c_-S | 1.01 | 0.95 – 1.08 | 0.38 | 0.704 | -0.01 | -0.03 – 0.01 | -1.27 | 8852.5 | 0.204 |
| conC_c_-S | 1.07 | 1.02 – 1.14 | 2.54 | **0.011** | 0.04 | 0.02 – 0.06 | 3.93 | 8853.5 | **<0.001** |
| conC_c_-C_p_ | 0.95 | 0.89 – 1.01 | -1.68 | 0.093 | -0.04 | -0.06 – -0.02 | -3.56 | 8848.9 | **<0.001** |
| conC_p_-S | 1.03 | 0.97 – 1.09 | 0.93 | 0.355 | 0.02 | 0.00 – 0.04 | 2.10 | 8851.7 | **0.036** |
| accuracy | 7.23 | 6.35 – 8.22 | 30.12 | **<0.001** | 0.41 | 0.40 – 0.43 | 43.71 | 8851.5 | **<0.001** |
| visC_c_-S:visC_c_-C_p_ | 1.07 | 1.00 – 1.14 | 2.11 | **0.035** |  |  |  |  |  |
| conC_c_-S:conC_c_-C_p_ | 1.10 | 1.04 – 1.16 | 3.51 | **<0.001** | 0.02 | 0.00 – 0.04 | 2.13 | 8851.3 | **0.033** |
| Random Effects |  |  |  |  |  |  |  |  |  |
| σ^2^ | 3.29 |  |  |  | 2.96 |  |  |  |  |
| ICC | 0.36 |  |  |  | 0.20 |  |  |  |  |
| Marg. R^2^ / Cond. R^2^ | 0.146 / 0.452 | |  |  | 0.177 / 0.341 | |  |  |  |
| Note. OR = Odds Ratio; Marg. R² = marginal R²; Cond. R² = conditional R²; ICC = intraclass coefficient; Std. β = standardized beta estimates; CI = confidence intervall; log(solution time) = natural logarithm of solution time; visCp-S = visual similarity between perceptual cue and solution; visCp-Cc =visual cue-cue similarity; visCc-S = visual similarity between conceptual cue and solution; conCc-S =conceptual similarity between conceptual cue and solution; conCc-Cp = conceptual cue-cue similarity; conCp-S = conceptual similarity between perceptual cue and solution. | | | | | | | | | |

**
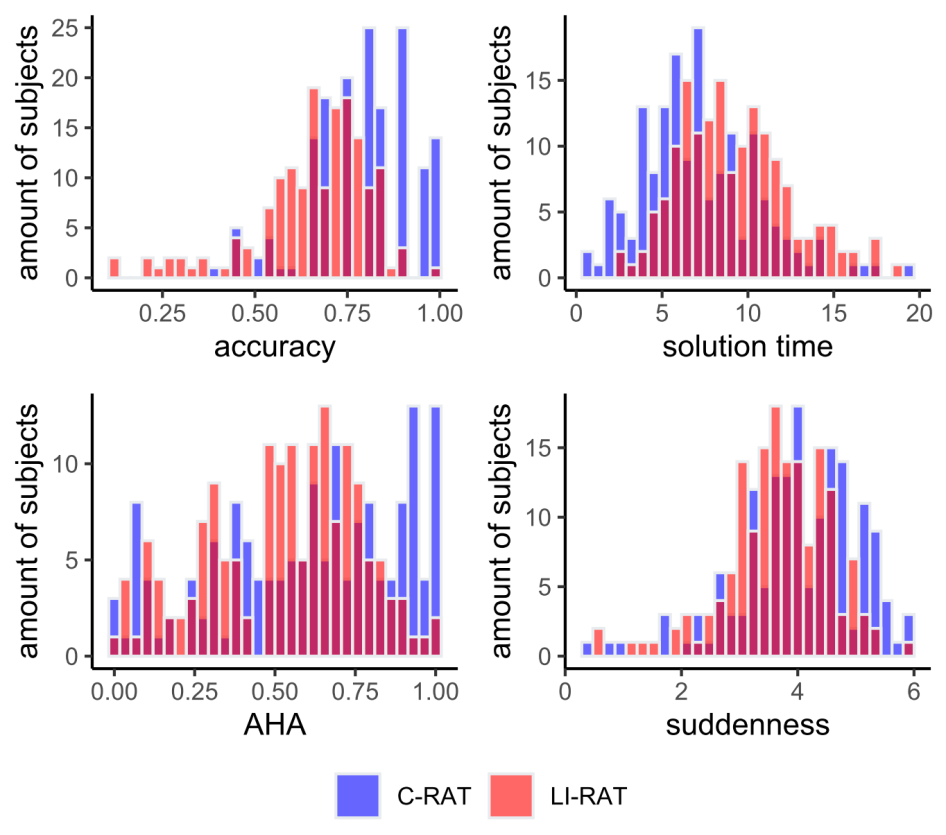
**

**Figure 4.** *Descriptive statistics of objective and subjective insight performance measures averaged per subject.*
